# Recent Advances in NADES-Assisted Process Intensification Technologies for Sustainable Recovery of Microalgal Bioactives: Challenges and Future Prospectives

**DOI:** 10.3390/md24040146

**Published:** 2026-04-21

**Authors:** Muhammad Shafiq, Sardar Ali, Liaqat Zeb

**Affiliations:** 1 School of Medical Sciences, Shandong Xiehe University, Jinan 250109, China; alisardar@sdxiehe.edu.cn; 2 Laboratory of Bioresources and Pharmaceutical Chemistry, Department of Chemistry, University of Bergen, 5007 Bergen, Norway

**Keywords:** microalgae, pre-treatment, bioactive compounds, natural deep eutectic, solvents, process-intensification technologies

## Abstract

Microalgae are increasingly recognized as renewable biofactories for producing high-value bioactive molecules. However, their industrial exploitation is limited by their rigid cell walls, metabolite heterogeneity, and the energy-intensive nature of the extraction processes. Recent advances in process-intensification technologies, including microwave-assisted, ultrasound-assisted, enzymatic, pressurized liquid, and supercritical CO_2_-based methods, have significantly improved extraction efficiency and selectivity, with reported lipid recoveries exceeding 40–50% in some microalgal systems and carotenoid recoveries approaching 90% under optimized conditions. NADES-assisted systems further enhance mass transfer and solubilization through tailored hydrogen-bonding interactions, enabling selective extraction of polar and semi-polar metabolites under mild conditions. However, limitations remain, including high viscosity, variability in extraction performance, and challenges in solvent recovery and scale-up. This review critically evaluates the extraction efficiency, mechanistic basis, and sustainability of NADES-assisted processes, highlighting key limitations and identifying research priorities for their integration into scalable microalgal biorefinery systems.

## 1. Introduction

The rising demand for sustainable, functional ingredients, coupled with escalating environmental pressures and finite natural resources, has intensified interest in alternative sources of bioactive compounds for the food, pharmaceutical, cosmetic, and nutraceutical industries [1]. Conventional crop-based systems are increasingly constrained by climate change, drought, limited arable land, and the environmental burden associated with pesticide use, prompting the search for more resilient and resource-efficient production platforms. In this context, microalgae have emerged as compelling alternatives to conventional energy sources [2,3].

Microalgae are fast-growing photosynthetic microorganisms capable of generating high biomass yields over short cultivation cycles and thriving in diverse environments, including saline and brackish waters and non-arable land [4,5]. Their ability to capture atmospheric CO_2_ and utilize nutrients from waste streams, combined with minimal freshwater requirements and the absence of herbicides or fungicides, positions microalgae as a cornerstone of future bioeconomy strategies. High areal productivity and operational flexibility further enhance their industrial relevance [4,5].

Microalgal biomass is a rich reservoir of structurally and functionally diverse bioactive compounds, including pigments (carotenoids, chlorophylls, and phycobiliproteins), polyunsaturated fatty acids, polysaccharides, proteins, and bioactive peptides [6,7,8]. Many of these metabolites exhibit antioxidant, anti-inflammatory, immunomodulatory, and lipid-regulating properties, and microalgae also provide substantial quantities of essential vitamins, often exceeding the levels found in conventional food sources. The global market for microalgal bioactives is expanding, driven by consumer demand for natural, plant-based, environmentally friendly products [9,10,11,12].

Despite this promise, the efficient recovery of microalgal metabolites remains a critical bottleneck. Rigid cell walls, high intracellular water content, and compartmentalized metabolite localization complicate extraction, necessitating effective pretreatment strategies to enhance solvent accessibility. Traditional solvent-based techniques, including Soxhlet extraction and maceration using organic solvents such as hexane, methanol, and chloroform, are energy-intensive, environmentally hazardous, and poorly suited for large-scale deployment [3,13].

Advanced extraction technologies, such as ultrasound-assisted, microwave-assisted, enzyme-assisted, pressurized liquid, and supercritical CO_2_ extraction, have substantially improved extraction efficiency and selectivity while reducing solvent consumption [14,15,16,17,18]. However, challenges related to compound specificity, environmental safety, and industrial scalability persist [3,19,20]. The integration of these techniques with green solvents (natural deep eutectic solvents (NADES)) offers a promising pathway to overcome these limitations [3,21]. Natural deep eutectic solvents (NADES), which are composed of biocompatible hydrogen-bond donors and acceptors derived largely from renewable resources, have emerged as leading candidates for sustainable solvent systems [3,22]. Their tunable polarity, adjustable viscosity, low volatility, and strong solvation capacity for polar and semi-polar compounds enable the tailored extraction of microalgal metabolites while facilitating downstream processing compatible with food and pharmaceutical applications [22,23].

Coupling NADES with advanced extraction technologies, such as ultrasound, microwave irradiation, or pressurized liquid extraction, has been associated with improved mass transfer, extraction efficiency, and energy economy [3]. The modularity of NADES-based systems further enables the selective fractionation of pigments, lipids, proteins, and polysaccharides from a single biomass stream under mild temperature and pressure conditions, reducing solvent inventories and simplifying purification workflows [3,24]. Representative applications include the extraction of carotenoids (e.g., astaxanthin, lutein), phenolic compounds, polysaccharides, and proteins using tailored NADES systems, demonstrating their versatility across diverse classes of microalgal bioactives [25]. However, despite these advances, the performance of NADES-based systems varies widely across studies due to differences in solvent composition, biomass characteristics, and experimental conditions, highlighting the need for critical evaluation of experimental design and reproducibility.

This review aims to critically evaluate sustainable extraction strategies for microalgal bioactive compounds, with particular emphasis on NADES-assisted processes, their extraction performance, mechanistic basis, and associated scalability and regulatory challenges. We summarize the key classes of microalgal bioactives, critically assess conventional and emerging extraction methods, discuss the physicochemical fundamentals of NADES, and examine representative case studies of NADES-assisted intensification technologies. Finally, we evaluate sustainability metrics, including life cycle and techno-economic assessments, and highlight the remaining challenges and future directions for integrating NADES into low-waste, circular biorefinery frameworks.

### Literature Search and Selection Strategy

A structured and transparent literature search was conducted using Scopus, Web of Science, and PubMed to identify relevant studies on microalgal bioactives, green extraction technologies, and Natural Deep Eutectic Solvents (NADES). The search covered publications from 2010 to 2025, with emphasis on recent developments in sustainable extraction approaches. Search queries combined keywords such as microalgae, bioactive compounds, NADES, deep eutectic solvents, green extraction, process intensification, ultrasound-assisted extraction, microwave-assisted extraction, supercritical CO_2_ extraction, and biorefinery. The initial search yielded approximately 554 articles. After removal of duplicates and screening based on titles and abstracts, approximately 373 studies were retained for full-text evaluation. Following detailed assessment against the inclusion criteria, a total of 264 articles were included in this review. Studies were included if they (i) focused on microalgal biomass or derived bioactive compounds, (ii) evaluated extraction processes with emphasis on green or intensified technologies, or (iii) investigated NADES systems in extraction applications. Studies were excluded if they were unrelated to extraction processes, focused solely on cultivation, lacked experimental or mechanistic detail, or were outside the scope of microalgal systems.

In addition to selection, studies were critically evaluated based on extraction performance, methodological rigor, reproducibility, solvent composition, and scalability potential. Particular emphasis was placed on studies providing comparative data, mechanistic insights, or process-level analysis relevant to industrial translation. Reference lists of selected articles were further screened to identify additional relevant studies. This approach ensures that the review not only summarizes existing studies, but also critically assesses their methodological robustness and practical relevance.

## 2. Bioactive Compounds in Microalgae

Microalgae exhibit exceptional taxonomic and metabolic diversity, making them a prolific source of bioactive compounds with broad industrial relevance and applications. This biochemical richness underpins their growing importance across multiple sectors, including food, cosmetics, aquaculture, biofuels and pharmaceuticals [7]. The ability of microalgae to thrive in highly variable aquatic environments has driven the evolution of diverse metabolic pathways, resulting in the accumulation of structurally and functionally distinct industrially relevant metabolites.

Microalgal biomass is a valuable reservoir of long-chain polyunsaturated fatty acids, sterols, proteins, polysaccharides, and phenolic compounds, among other bioactives (Table 1). These compound classes arise from distinct biosynthetic routes and cellular processes, collectively providing a versatile biochemical platform for industrial applications. The functional diversity of these metabolites supports applications ranging from nutrition and health to energy and materials, reinforcing the commercial and biotechnological significance of microalgae as sustainable resources.

From a chemical perspective, these bioactive compounds can be classified based on their structural characteristics, including polarity, functional groups, and molecular architecture, which directly influence their extractability and interaction with solvent systems such as NADES.

### 2.1. Pigments: Carotenoids, Chlorophylls and Phycobiliproteins

**Figure 1:**
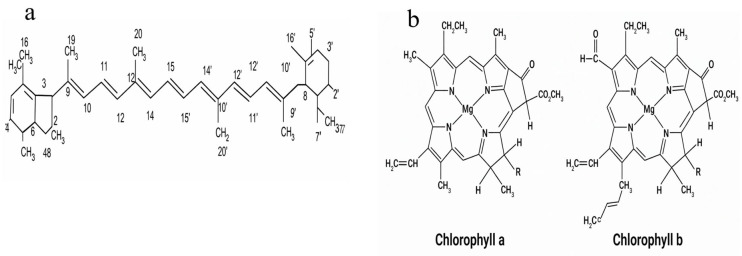
Representative chemical structures of microalgal pigments, including (**a**) carotenoids (**b**) chlorophylls.

Among microalgal bioactives, pigments represent one of the most commercially advanced classes, underpinning both research and industrial applications of microalgae [58]. The primary pigment groups chlorophylls, carotenoids, and phycobiliproteins dominate microalgal photophysiology and account for the majority of pigment-derived products [58]. From a chemical perspective, these pigments differ significantly in molecular structure, polarity (Figure 1), and stability, which directly influence their extraction behavior and solvent compatibility.

Chlorophylls, the central pigments of photosynthetic organisms, are abundant intracellularly and have attracted increasing interest as natural colorants due to their antioxidant and detoxifying properties [59,60]. Structurally, chlorophylls consist of porphyrin-like macrocycles coordinated with a central magnesium ion and a hydrophobic phytol tail, which governs their amphiphilic nature and sensitivity to environmental conditions. Species of the genus *Chlorella*, in which chlorophyll can constitute up to ~7% of the biomass, are widely exploited for commercial production. However, chlorophyll integrity during extraction remains a critical limitation, as these molecules are highly susceptible to thermal degradation, pH fluctuations, and oxidative stress [59,60].

Carotenoids constitute the second major pigment class and include β-carotene, lutein, zeaxanthin, fucoxanthin, and the high-value ketocarotenoid astaxanthin. These compounds are characterized by extended conjugated polyene chains, which determine their strong lipophilicity and susceptibility to oxidation. Their biosynthesis via isoprenoid pathways is tightly regulated by light intensity and nutrient stress, which is exploited in industrial cultivation systems [61,62]. *Haematococcus pluvialis* is the dominant platform for natural astaxanthin production, while species of *Chlorella*, *Muriellopsis*, and marine diatoms are cultivated for lutein and fucoxanthin, supporting the expanding food and nutraceutical markets [63,64,65,66].

Phycobiliproteins, including phycocyanin and phycoerythrin, are water-soluble protein pigment complexes characteristic of cyanobacteria. Structurally, they consist of covalently bound tetrapyrrole chromophores attached to specific apoproteins, which confer high solubility but also sensitivity to environmental stress. C-phycocyanin derived from Arthrospira has achieved commercial relevance as a natural colorant, fluorescent probe, and bioactive compound [58,67]. However, extraction and purification remain challenging due to protein denaturation and pigment instability [68,69].

From a bioprocessing perspective, pigment extraction strategies must be aligned with their structural and physicochemical properties. Lipophilic carotenoids favor non-polar or hydrophobic NADES systems, whereas phycobiliproteins require mild aqueous environments to preserve protein structure. Examples of hydrophobic NADES include menthol–decanoic acid and menthol–lauric acid systems, which exhibit low polarity and are particularly suitable for extracting lipophilic compounds such as carotenoids. Given their sensitivity to oxygen, pH, light, and temperature, optimized cultivation, harvesting, extraction, and formulation strategies are essential to minimize degradation and maintain bioactivity [22,58]. Certain NADES systems have been reported to enhance pigment stability by reducing oxidative degradation; however, highly acidic or high-viscosity systems may negatively affect pigment integrity, emphasizing the need for composition-specific optimization [70].

### 2.2. Lipids and Long-Chain Poly-Unsaturated Fatty Acids (PUFAs)

**Figure 2:**
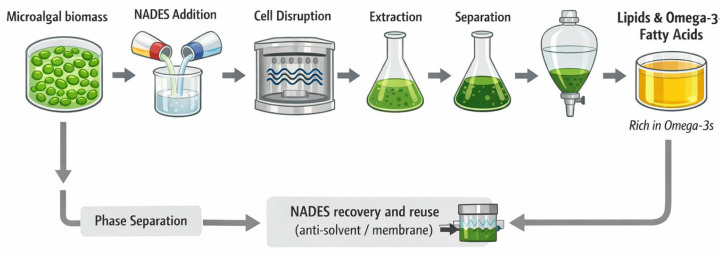
NADES-assisted extraction of omega-3 fatty acids from oleaginous microalgae, including solvent recovery and reuse.

Microalgae are widely recognized as a sustainable and scalable source of lipids and long-chain polyunsaturated fatty acids (LC-PUFAs), offering a compelling alternative to fish-derived oils. Among these, the omega-3 fatty acids eicosapentaenoic acid (EPA) and docosahexaenoic acid (DHA) are of particular industrial relevance, underpinning applications across the food, nutraceutical, and pharmaceutical sectors (Figure 2) [71,72]. Unlike terrestrial oilseed crops, many microalgal taxa synthesize EPA and DHA de novo and accumulate them in both neutral lipids, such as triacylglycerols, and polar lipid fractions, including phospho- and glycolipids, which have important implications for bioavailability and functional performance [71,72]. These lipids are distributed across neutral lipid fractions, such as triacylglycerols, and polar membrane lipids, including phospholipids and glycolipids, which differ in polarity and influence solvent selection during extraction. Several genera, including *Nannochloropsis*, *Tetraselmis*, *Schizochytrium*, and *Chlorella*, are established producers of EPA and DHA, whereas marine cryptophytes exhibit some of the highest natural omega-3 densities reported to date nature [73]. The distribution of PUFAs across lipid classes is species- and condition-dependent, with membrane lipids, particularly phosphatidylethanolamines and phosphatidylglycerols, often showing pronounced enrichment [74].

The nutritional and therapeutic significance of microalgal omega-3 fatty acids has been well documented. EPA and DHA contribute to cardiovascular health, lipid homeostasis, and immune regulation, and DHA is a structural component of neuronal membranes which is essential for visual and neurocognitive functions. Deficiency in omega-3 PUFAs, particularly during pregnancy and early development, has been associated with impaired sensory function and altered immune maturation, whereas supplementation can support immune development and reduce the risk of allergic diseases in early life [75]. Beyond systemic effects, omega-3 lipids influence the gut microbiota composition and intestinal barrier integrity and interact with bioactive mediators, such as short-chain fatty acids and endocannabinoids [75].

Commercially, microalgal EPA and DHA oils are increasingly used as sustainable alternatives to fish and vegetable oils, reducing the pressure on marine ecosystems while enabling the production of highly purified omega-3 concentrates [76]. In addition to PUFAs, algal lipid fractions contain sterols, tocopherols, and carotenoids, which may exert synergistic effects in formulated products. Traditionally, lipid recovery relies on solvent-based methods such as Bligh–Dyer or Folch extraction [77,78]. However, greener approaches, including supercritical CO_2_ and NADES-assisted extraction, are gaining prominence due to their improved selectivity, oxidation control, and compliance with food-grade and regulatory requirements [79,80].

### 2.3. Proteins and Bioactive Peptides

Microalgae are a rich and increasingly important source of proteins and bioactive peptides, with several taxa, most notably *Arthrospira* and *Chlorella*, achieving protein contents exceeding 40–60% of dry biomass under optimized cultivation conditions [81,82]. Microalgal proteins typically provide a complete essential amino acid profile and, upon enzymatic hydrolysis, yield a diverse array of bioactive peptides with antihypertensive, antioxidant, anti-inflammatory, immunomodulatory, and metabolic regulatory activities [81,83]. These properties have led to a growing interest in microalgal proteins and peptides as functional food ingredients and therapeutic leads [84,85,86].

Among the most extensively studied bioactivities are angiotensin-converting enzyme (ACE)-inhibitory and antioxidant peptides, which have been associated with cardiovascular risk reduction, improved glucose homeostasis, and attenuation of chronic inflammatory responses [85,87]. Advances in molecular biology, peptidomics, in silico digestion models, and bioinformatics have substantially accelerated the identification and prediction of bioactive peptide sequences from complex microalgal proteomes, enabling targeted discovery and functional screening of bioactive peptides.

**Figure 3:**
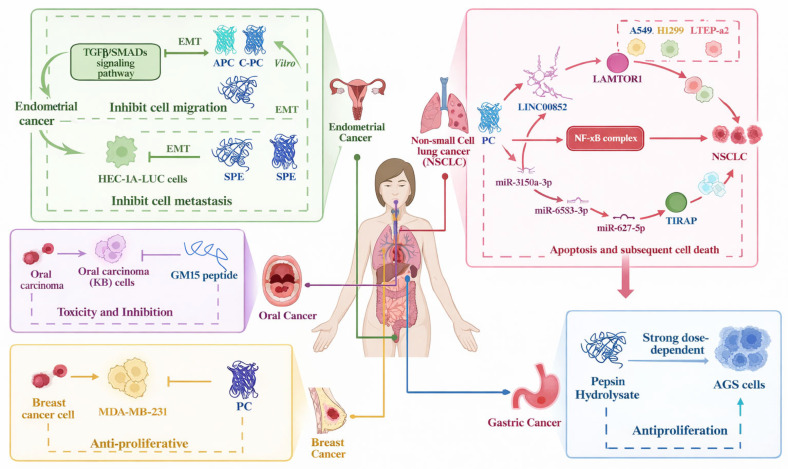
The anticancer mechanisms associated with microalgal proteins (MPs), protein hydrolysates (MPHs), and protein-derived peptides (MPPs). Arrows terminating in a perpendicular bar indicate inhibitory effects. Adapted with permission from [88].

Beyond cardiometabolic health, microalgal-derived peptides have attracted attention for their potential anticancer activity. Diet-related factors contribute significantly to cancer risk, and several studies have reported that algal peptides can modulate apoptosis, regulate cell-cycle progression and counteract oxidative stress through defined molecular pathways. These mechanisms may enable selective inhibition of cancer cell proliferation while reducing the systemic toxicity associated with conventional treatments such as chemotherapy and radiotherapy (Figure 3) [88]. Figure 3 summarizes the key molecular mechanisms through which microalgal-derived peptides exert anticancer effects, including inhibition of cell proliferation, induction of apoptosis, and modulation of signaling pathways.

Despite their considerable promise, the efficient recovery of microalgal proteins and peptides remains a significant technical challenge. The rigid, polysaccharide-rich cell walls of microalgae restrict protein release, necessitating mechanical, enzymatic, or combined disruption strategies [89]. In addition, protein integrity is highly sensitive to extraction conditions, including pH, ionic strength, and temperature, requiring careful process control. For peptide production, controlled enzymatic hydrolysis coupled with targeted fractionation is essential to preserve bioactivity while minimizing non-specific proteolysis [90]. These constraints highlight the need for judicious selection of pretreatment methods, solvents, and green extraction technologies to enable the sustainable recovery of high-value proteinaceous products.

### 2.4. Polysaccharides and Sulfated Exopolysaccharides

Interest in microalgal polysaccharides has grown substantially in recent years owing to their structural diversity and broad spectrum of biological activities, attracting increasing attention from the food, pharmaceutical, and biomedical sectors [91]. These polymers exhibit distinctive molecular features, including branching architecture, degree of polymerization, sulfation patterns, and molecular weight distribution, which underpin their functional and bioactive properties. Their natural origin, biodegradability, and favorable safety profiles further position microalgal polysaccharides as attractive candidates for medical and nutraceutical applications [92,93,94].

The bioactivity of microalgal polysaccharides is closely linked to their chemical composition, which often includes uncommon sugar residues, uronic acids and sulfate groups. These negatively charged moieties confer unique conformational and physicochemical properties that govern biological function, such as the dependence of antiviral activity on sulfation degree and pattern [94,95,96]. Examples include, polysaccharides from *Euglena gracilis* (EgPs), characterized by FT-IR and GC–MS, showed low cytotoxicity toward normal HGF-1 cells (IC_50_ = 228.66 µg/mL) and strong anticancer activity, particularly against HCT-116 colon cancer cells (IC_50_ = 26.1 µg/mL) [97]. Naviculan, a sulfated polysaccharide isolated from the diatom *Navicula directa* demonstrated strong antiviral activity against HSV-1, HSV-2, and influenza A, with high selectivity indicating effective and safe inhibition. It also inhibited HIV-related cell–cell fusion, highlighting its potential as a broad-spectrum antiviral compound [98]. In food applications, microalgal polysaccharides serve as stabilizers, texture modifiers and gelling agents, enhancing product structure and consumer appeal, particularly in confectionery and dessert formulations. Their role as dietary fiber further supports gut health and digestive function, reinforcing their suitability for functional foods and dietary supplements [94].

The extraction and purification of microalgal polysaccharides typically involve aqueous extraction, followed by selective precipitation, dialysis, and chromatographic fractionation based on size and charge. Recovery is frequently complicated by the co-extraction of proteins, salts, and pigments, necessitating multistep purification workflows [99,100]. Recent advances focus on integrating green solvents, membrane-based purification, and process intensification techniques to improve yield, selectivity, and scalability while maintaining structural integrity [101].

### 2.5. Phenolics and Other Secondary Metabolites

Microalgae are a rich and underexplored source of phenolic compounds and other secondary metabolites with significant functional and biomedical relevance. Microalgal phenolics exhibit diverse bioactivities, including antioxidant, antimicrobial, and anti-inflammatory effects, underpinning their value in the food and pharmaceutical industries. The marine origin of many microalgal species contributes to their structurally distinctive phenolic profiles, which may enhance their biological efficacy and expand their potential applications [102,103].

Phenolic compounds identified in microalgae include phenolic acids, flavonoids, and other aromatic metabolites that function as antioxidants through free radical scavenging and metal chelation mechanisms, and as allelochemicals in aquatic ecosystems [102,103]. These activities contribute to the mitigation of oxidative stress, a key factor implicated in cancer development and cardiovascular diseases. Accordingly, microalgal phenolics have attracted interest for reducing disease risk and as natural antioxidants in food systems to prevent lipid oxidation and extend the shelf life [103,104]. Phenolic content varies widely among species and is influenced by cultivation conditions and analytical parameters, including extraction solvents, methodologies, and environmental stressors [103]. Identified compounds include gallic acid, caffeic acid, ferulic acid, and flavonoids such as quercetin and catechin, which contribute to antioxidant and antimicrobial activity [102,105]. Despite their demonstrated bioactivity, the large-scale commercialization of microalgal phenolics remains limited. Regulatory barriers and an incomplete understanding of their safety profiles and in vivo efficacy necessitate further investigation to support their translational and commercial applications [106]. Regulatory barriers include the lack of standardized approval frameworks for novel microalgal compounds, limited toxicological data, and restrictions related to solvent residues in food and pharmaceutical applications [106].

Beyond phenolics, microalgae produce a broad array of secondary metabolites, including alkaloid-like compounds, terpenoids, lectins, sulfated peptides, and halogenated molecules such as furanones and depsipeptides [107]. Many of these compounds exhibit antimicrobial, cytotoxic, or pharmacologically relevant activities and are of growing interest in the pharmaceutical sector. The vast taxonomic diversity of microalgae, coupled with the large number of uncharacterized species, highlights their potential as reservoirs for novel natural products [108].

However, the isolation and purification of these structurally complex metabolites remains technically challenging, often limiting their commercial exploitation. Recent advances in green extraction and downstream processing technologies have improved the recovery efficiency and compound integrity, offering more sustainable, scalable, and economically viable routes for harnessing microalgal secondary metabolites [103].

## 3. Conventional Extraction Approaches and Their Limitations in Microalgae Biorefinery

### 3.1. Classical Solvent-Based, Thermal, and Mechanical Methods

The selection of extraction strategies for microalgal bioactive compounds is critical for downstream industrial applications. Historically, the recovery of microalgal metabolites has relied on classical solvent-based methods that employ organic solvents with recognized environmental and health issues. Common approaches include maceration, Soxhlet extraction, and lipid-focused protocols such as the Bligh–Dyer and Folch methods.

Maceration is a simple solid–liquid extraction technique driven by passive diffusion and is widely used because of its simplicity. However, its long extraction times, low mass transfer efficiency, and poor performance with low-polarity compounds or complex matrices limit its applicability, particularly for microalgae with rigid and chemically resistant cell walls [109]. To overcome these constraints, maceration is often combined with mechanical pretreatments, including bead milling (a mechanical disruption technique using high-speed agitation of solid beads to rupture cell walls), high-pressure homogenization, or freeze–thaw cycles, to disrupt cellular barriers and enhance solvent penetration. These additional steps, along with high solvent requirements, increase operational costs and environmental burden [109,110].

Soxhlet extraction employs continuous solvent reflux and condensation to achieve an exhaustive extraction. Although effective, this method is characterized by prolonged processing times, high energy demand, and substantial solvent consumption. Extended thermal exposure further increases the risk of degrading thermolabile bioactive compounds, restricting their suitability for high-value applications [110,111]. Similarly, the Bligh–Dyer and Folch protocols are widely used for lipid extraction, relying on chloroform–methanol systems to partition lipids into the organic phase. Despite their effectiveness, these methods require biomass drying, large volumes of halogenated solvents, and additional cell disruption steps, resulting in labor-intensive workflows and significant environmental and safety concerns.

Overall, although classical solvent-based and mechanical extraction methods remain prevalent, they are fundamentally limited by low selectivity, inefficient mass transfer, and dependence on hazardous solvents. In practice, these limitations necessitate supplementary mechanical pretreatments to partially offset the structural and kinetic constraints, underscoring the need for more efficient and sustainable extraction alternatives.

### 3.2. Environmental, Regulatory, and Operational Constraints

Although conventional extraction methods remain widely used, they are increasingly regarded as unsustainable when assessed against contemporary regulatory frameworks and green chemistry principles. A primary concern is their reliance on large solvent volumes, extended processing times, and high energy inputs, together with the use of hazardous organic solvents such as chloroform, hexane, and dichloromethane, which pose substantial environmental and health risks [112,113].

Despite growing awareness, methanol-, chloroform- and hexane-based systems are still frequently employed in microalgal extraction. These solvents are flammable, environmentally unfavorable and potentially toxic, and their use raises significant regulatory challenges. Residual solvent contamination is a critical issue, particularly for extracts intended for food, nutraceutical or cosmetic applications, where trace levels of halogenated or non-food-grade solvents can impede regulatory approval and market adoption [114]. Strategies to mitigate residual solvent contamination include vacuum evaporation, membrane separation, adsorption techniques, and solvent-switching approaches to ensure compliance with regulatory limits [115].

Energy intensity is another limitation. Conventional extraction often requires biomass drying prior to processing, followed by solvent heating, circulation, and evaporation, all of which substantially increase energy demand [111]. In lipid recovery from microalgae, extraction and associated drying steps can account for up to 90% of the total process energy consumption. Prolonged exposure to elevated temperatures and oxidative conditions further increases the risk of degrading thermolabile compounds, including carotenoids, polyunsaturated fatty acids, and phycobiliproteins, thereby reducing the yield and altering bioactivity [116,117].

From an operational perspective, classical methods typically exhibit low selectivity, resulting in the co-extraction of undesired components, such as chlorophyll degradation products, free fatty acids, and residual solvents. These impurities complicate downstream purification, increase solvent recovery requirements, and escalate processing costs, particularly at scale.

Finally, the environmental footprint of conventional extraction processes is substantial. Many commonly used solvents are volatile organic compounds (VOCs), necessitating specialized waste handling and recovery systems and contributing to emissions and hazardous waste generation. The large-scale industrial use of such solvents has been linked to significant volumes of toxic chemical waste, raising compliance challenges as regulatory standards and consumer expectations increasingly favor clean-label, low-residue, and environmentally sustainable products [118]. A summary of representative microalgal species, their bioactive compounds, and applications is provided in Table 1.

### 3.3. The Imperative for Greener and Scalable Extraction Routes

The combined operational, environmental, and safety limitations of classical extraction methods underscore the urgent need for greener, more efficient, and industrially scalable alternatives. Next-generation extraction processes must minimize solvent consumption, replace hazardous chemicals with benign or bio-derived media, reduce energy demand through shorter processing times and lower operating temperatures, and deliver higher selectivity and yields to ease downstream purification requirements (Table 2) [119].

This transition is reflected in the rapid uptake of process-intensification technologies, including ultrasound-assisted extraction (UAE), microwave-assisted extraction (MAE), pressurized liquid extraction (PLE), supercritical CO_2_ extraction (SFE), and emerging bio-based solvent systems such as Natural Deep Eutectic Solvents (NADES). Comparative studies consistently demonstrate the superior performance of these approaches relative to conventional techniques; for example, SFE-CO_2_ has achieved lipid recoveries of approximately 45% from *Nannochloropsis* species, exceeding yields obtained using Soxhlet or Bligh–Dyer extraction under comparable conditions [119,120,121]. However, beyond extraction efficiency, green technologies must satisfy additional criteria to enable industrial deployment, including scalability, solvent recyclability, compatibility with upstream cultivation and downstream purification, and favorable techno-economic performance. As the microalgal sector increasingly aligns with circular and bio-based production paradigms, extraction routes that minimize or eliminate organic solvents and can be integrated into closed-loop, energy-efficient biorefinery systems are gaining priority [122]. Accordingly, the shift from conventional to sustainable extraction strategies is not merely advantageous but essential for the environmentally and economically viable production of microalgal bioactive compounds on a large scale.

**Table 2:** Summary of the extraction yield of microalgal bioactive compounds through advanced green extraction technologies.

Microalgal Species	Cascade/Extraction Workflow	Target Compound(s)	Reported Metric(s)	Reference
*Spirulina platensis*	Ultrasound-assisted extraction (UAE) using a NADES (optimized by RSM)	total pigments, total phenolics,	Temperature: 40–60 °C total pigment yield = 165.19 ± 1.01 mg·g^−1^ dry matter; total phenolic content = 36.50 ± 0.98 mg GAE·g^−1^ DM;	[123]
*Haematococcus pluvialis*	Supercritical CO_2_ extraction (SFE) with ethanol co-solvent (process optimization)	astaxanthin and lutein	Temperature: 50 °C, Pressure: 550 bar, CO_2_ flow 3.62 g·min^−1^. gave astaxanthin recovery up to 98.6%; lutein recovery up to 52.3%.	[124]
*Haematococcus pluvialis*	SFE kinetics study (effect of CO_2_ flow rate, temp, pressure)	astaxanthin	Temperature: 50 °C Pressure: 50 MPa	[125]
*Nannochloropsis* sp.	Supercritical CO_2_ extraction	total lipids and lipid-class distribution (neutral lipids, FFA, acylglycerides)	Pretreatment doubled total extraction yield, reaching 23.1 wt% (comparable to 26.9 wt% by Bligh–Dyer); pretreatment also nearly doubled free fatty acids and increased acylglycerides	[126]
*Phaeodactylum tricornutum*	Supercritical fluid extraction (SFE)	fucoxanthin	Temperature: 30 °C Pressure: 30 MPa Solvent: 40% ethanol. Highest extract yield = 24.41% (*w*/*w*) at; highest fucoxanthin purity = 85.03 mg fucoxanthin per g extract and recovery = 66.60% *w*/*w*	[127]
*Ascophyllum nodosum*	Subcritical water extraction	polysaccharide	160 °C/18 min/0.1 g mass. Yield Alginate: 38.25 dry wt% Fucoidan: 39.38 dry wt%	[14]
*Spirulina platensis*	Coupling of ultrasound and subcritical water extraction	bioactive extract peptides production from Spirulina platensis	Ultrasound (power 221 W, time 64 min) + (subcritical water 153 °C), Protein extraction rate: 67.10 ± 0.004%	[128]
*Chlorella pyrenoidosa*	Subcritical water extraction	total lipids, carbohydrate, protein	270 °C/10 min/15% (*w*/*v*), oil yield of 12.89 wt%, (*w*/*v*), Total protein, 4.13 mg/mL, Carbohydrate: 1.35 mg/mL	[129]
*Anabaena planctonica*	Pressurized liquid extraction (PLE)	total lipids for biodiesel production	Temperature: 200 °C Pressure: 20.7 MPa Time: 15 min Solvent: Limonene: ethanol (1:1). total Yield (3.1 ± 0.5% *w*/*w*) Lipids (6.0 ± 1.1% *w*/*w*) Gamma-linolenic acid (1.3 ± 0.4% *w*/*w*)	[130]
*Spirulina*, *Chlorella*, and *Phaeodactylum tricornutum*	Microwave-assisted extraction (MAE)	carotenoids polyphenols, chlorophyll	T: 40 °C P: 103.4 bar Time: 15 min Solvent: DMSO (100%) Magnetic stirring. polyphenols 10.465 mg/g, chlorophyll a 6.206 mg/g, chlorophyll b 3.003 mg/g, carotenoids 0.971 mg/g	[131]
*Arthrospira platensis*	Enzyme-Assisted Extraction (EAE)	Allophycocyanin	Ultrasonication Enzyme: Lysozyme Temperature: 27 ± 2 °C pH: 6.8 Time: 4 h Intermittent stirring	[132]
*Chlorella pyrenoidosa*	Supercritical fluid extraction (SFE) SFE-CO_2_	chlorophyll a chlorophyll b lutein β-Carotene	Temperature: 60 °C Pressure: 30 MPa Time: 120 min Co-solvent: Ethanol 7.5% (*v*/*v*). Chlorophyll a (37.50%) Chlorophyll b (2.83%) Lutein (19%) β-Carotene (2.93%)	[133]
*Chlorella pyrenoidosa*	Microwave-assisted extraction (MAE)	total lipids	Temperature: 60 °C Pressure: - Time: 5 min Power: 700 W Solvent: 2.5% of hydrophilic Ionic Liquids	[134]
*Galdieria phlegrea*	Pressurized liquid extraction (PLE)	carotenoids	Temperature: 50 °C Pressure: 100 bar Time: 30 min Solvent: Ethanol. Carotenoids (89 ± 6 mg/g dw)	[135]
*Nannochloropsis gaditana*	Supercritical fluid extraction (SFE-CO_2_)	proteins, carbohydrates, lipids, SFAs MUFAs, lipids EPA, Other PUFAs	Temperature: 50 °C Pressure: 350 bar Time: 135 min. Proteins (42.73% dw) Carbohydrates (27.67% dw) Lipids (11.83% dw) SFAs (32.20% lipids dw) MUFAs (27.21% lipids dw) EPA (33% lipids dw) Other PUFAs (7.59% lipids dw)	[136]
*Chlorella vulgaris and Porphyridium purpureum*	Ultrasound-assisted extraction (UAE)	carotenoids, Lipids	Temperature: room temperature Time: 30 min Power: 70% Solvent: 70% Ethanol/hexane 2:1 (*v*/*v*) Carotenoids (947.25 µg/g dw) Lipids (21.59 mg/g dw)	[137]
*Haematococcus pluvialis* (*H. Pluvialis*)	Ultrasound-assisted extraction (UAE)	carotenoids	Temperature: 50 °C Power: 648 W Solvent: acetone, D-limonene, and medium chain triglycerides (MCT) oil at the ratios, 1:100, 1:50, 1:20, 1:10, and 1:5 g biomass/mL solvent.	[138]
*Coccomyxa onubensis*	Supercritical fluid extraction (SFE)	carotenoids total phenols	Temperature: 70 °C Pressure: 55 MPA bar Time: 15 min. Solvent: (0, 25%, and 50% *v*/*v* ethanol). Yield of total phenols 66.98%	[139]
*Porphyridium purpureum*	Ultrasonic (UAE) and microwave-assisted (MAE)	lipid and carbohydrate	Power: 30% Time: 20 min Solvent: MeOH-CHCl_3_. recovery of 69% lipid and 57% carbohydrate	[140]
*Chlorella* sp.	Ultrasound-assisted extraction (UAE)	lipids	Temperature: 60 °C Time: 25 min Power: 40 kHz Solvents: ethanol–2-MeTHF mixture (2:1, *v*/*v*) lipid yield of 25.05 ± 0.924%	[141]
*Auxenochlorella protothecoides*	Pulsed microwave (PMW)	lipids	Temperature: 70 °C Time: 30 min, Pulse: 100 to 300 μs Power: 2 and 4 kW Lipids yield 38.42%	[142]
*Chlorella vulgaris* and *Nannochloropsis oculata*	Ultrasound-assisted extraction (UAE)	lipids	Power: 80 kHz Times: 20 min lipid yield 11.54% for *Chlorella vulgaris* and 17.91% for *Nannochloropsis oculata*	[143]
*Chlorella vulgaris*	Enzyme-assisted Extraction (EAE).	bioactive peptides	Enzymes: Proteases and cellulase, Incubation: protease hydrolysis temperature of 50 °C and 1.67% protease for 2 h. Solvent: 2% acetic acid solution	[144]
*Chlorella pyrenoidosa*	Ultrasound-assisted extraction (UAE)	protein	Temperature: 60 °C Power: 10 W, 200 W, and 700 W. Maceration extraction: 300 rpm of agitation. 9-time protein yields and 3-time extraction rate compared to conventional solvent extraction Ultrasonic powers: 10 W, 200 W, and 700 W; Maceration extraction: 300 rpm of agitation at 60 °C	[145]

## 4. Natural Deep Eutectic Solvents (NADES): Fundamentals and Design for Microalgae Bioactive Recovery

Natural deep eutectic solvents (NADES) are liquid eutectic systems formed by combining two or more naturally derived components, typically a hydrogen-bond acceptor (HBA) and a hydrogen-bond donor (HBD), which establish extensive hydrogen-bond networks, resulting in melting points far below those of the individual constituents [146]. A representative example is the choline chloride–glycerol system (1:2), which forms a stable liquid at room temperature, although glycerol is already liquid under ambient conditions, and the eutectic interaction with choline chloride significantly modifies the physicochemical properties of the system [147,148].

**Figure 4:**
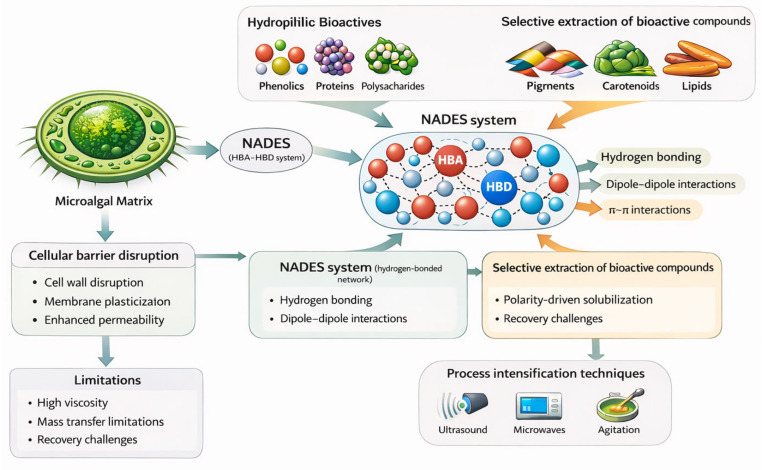
Mechanistic representation of NADES-assisted extraction of microalgal bioactive compounds, illustrating cellular disruption, hydrogen-bond-driven interactions, and polarity-dependent solubilization leading to selective extraction of hydrophilic and lipophilic compounds.

In green extraction, NADES function as tunable designer solvents, whose polarity, viscosity, and solvation capacity can be precisely adjusted through the rational selection of HBA/HBD pairs, molar ratios, and controlled water addition (Table 3). This compositional flexibility enables the selective solubilization of a broad spectrum of microalgal metabolites, ranging from highly polar polysaccharides and proteins to moderately nonpolar pigments and lipids (Figure 4). Because their constituents are typically natural metabolites, such as sugars, amino acids, organic acids, and choline derivatives, NADES offer a biocompatible and sustainable alternative to conventional organic solvents [146].

### 4.1. Types and Compositional Families

NADES can be categorized into compositional families based on the chemical nature of their HBAs and HBDs, and are broadly classified as hydrophilic or hydrophobic systems. NADES systems are commonly classified into four types (Type I–IV) based on the nature of the hydrogen bond acceptors and donors, including metal salt-based, quaternary ammonium salt-based, and organic acid-based systems. Hydrophilic NADES are commonly formulated from choline chloride paired with sugars, polyols, or organic acids, producing eutectic mixtures with moderate to high polarity at molar ratios typically ranging from 1:1 to 1:3. Amino acid-based NADES further expand this class by offering intermediate polarity combined with high biocompatibility [152].

Hydrophobic NADES, by contrast, are formed by combining weakly polar HBDs such as menthol or camphor with fatty acids (e.g., decanoic or lauric acid), yielding low-polarity liquids well suited for lipophilic solutes. Fine-tuning the molar ratio modulates the melting point, viscosity, mass transfer behavior, and overall extraction performance across both families [146,147].

### 4.2. Physicochemical Properties Influencing Extraction

The suitability of NADES for microalgal extraction is governed by key physicochemical parameters, including polarity, viscosity, water content, melting point, and solvation mechanisms. Hydrophilic NADES containing choline chloride and sugar or polyol donors exhibit dense hydrogen-bonding networks and high dielectric constants, facilitating the efficient solvation of polar metabolites such as proteins, peptides, polysaccharides, and phenolic compounds [153]. These systems can also enhance the solubility of poorly water-soluble flavonoids through cooperative hydrogen bonding, dipole–dipole interactions, π-π stacking, and ionic coordination, collectively disrupting solute-matrix interactions and stabilizing bioactives within the eutectic medium [153,154].

High viscosity remains a major limitation of many NADES, often exceeding 100 mPa·s at ambient temperatures, which can restrict diffusion and mass transfer. Partial hydration through the addition of 10–25 wt% water effectively reduces the viscosity, often by more than half while preserving the eutectic microstructure and extraction efficiency. Therefore, the water content plays a critical role in balancing the solvent mobility and solvation strength [70]. Recent studies on lactic acid-based NADES have demonstrated that water content significantly influences physicochemical properties, including viscosity, polarity, and antimicrobial activity. Increased water content reduces viscosity and enhances mass transfer, while simultaneously affecting hydrogen-bond interactions and extraction selectivity [155].

NADES typically exhibit low melting points and remain liquid at or below room temperature, reflecting strong hydrogen bond interactions and lattice energy depression. Many systems are thermally stable up to ~160 °C, enabling compatibility with process-intensified techniques such as microwave-assisted and pressurized-liquid extraction. For example, choline chloride–glycerol and choline chloride–lactic acid systems exhibit thermal stability up to ~150–160 °C [156]. These mild thermal characteristics are particularly advantageous for recovering thermolabile microalgal pigments [157].

At the molecular level, the HBA-HBD hydrogen-bond network governs the solvent microstructure, dielectric environment, and selectivity. The chemical nature of the donor groups (e.g., -OH, -COOH, and -NH_2_) influences network flexibility and mobility, directly affecting extraction behavior. For instance, NADES featuring localized nonpolar microdomains combined with moderate polarity have shown enhanced performance for carotenoid solubilization compared to highly polar solvents [158].

From a toxicological perspective, the safety of NADES-based extraction systems requires careful consideration, particularly for applications involving direct human exposure, such as food, nutraceutical, and cosmetic formulations [159,160]. Although many NADES components, including choline chloride and glycerol, are individually classified as Generally Recognized as Safe (GRAS), the formation of eutectic systems results in new hydrogen-bonded networks with distinct physicochemical properties that may alter biological interactions. These interactions can influence membrane permeability, compound solubility, and metabolic behavior, potentially leading to effects that are not predictable from the properties of the individual components [159,160]. Importantly, in cases where NADES are not removed during downstream processing and remain in the final product, their potential impact on human health must be evaluated at the system level. Emerging studies suggest that NADES toxicity is highly dependent on composition, concentration, pH, and water content, with certain formulations showing cytotoxic or inhibitory effects under specific conditions [161]. In addition, the high viscosity and strong solvation capacity of NADES may influence the bioavailability of co-extracted compounds, further complicating safety assessment [161].

Therefore, while NADES offer advantages as green solvents, their application in food and cosmetic systems requires cautious evaluation, including toxicological testing, dose assessment, and regulatory validation [162]. Current evidence highlights the need for systematic, case-by-case assessment of NADES formulations rather than assuming safety based solely on their natural origin or constituent components.

### 4.3. Synthesis, Characterization, and Practical Formulation Guidelines

NADES are most commonly prepared by heating and continuously stirring the HBA and HBD components at 50–80 °C until a homogeneous liquid is obtained, followed by mild vacuum drying to remove residual moisture. For thermally sensitive components or to fine-tune the viscosity and water content, alternative methods such as grinding, freeze-drying, vacuum evaporation, or ultrasonic mixing may be employed [147,158].

Comprehensive characterization is essential to ensure solvent reproducibility and enable mechanistic interpretation. Hydrogen-bond formation can be probed by FTIR spectroscopy through shifts in the -OH, -NH, and -CO stretching bands, while ^1H/^13C NMR spectroscopy provides insight into solute-solvent interactions and microstructural organization. Differential scanning calorimetry (DSC) can be used to quantify the melting point depression and thermal transitions, and rheological measurements can be used to define the viscosity and flow behavior, which critically influence the extraction kinetics. Advanced techniques, including dielectric spectroscopy and molecular dynamics simulations, have further elucidated nanoscale solvent organization and hydrogen bond dynamics [154,163,164].

Effective NADES design for microalgal extraction requires consideration of several practical criteria: (i) viscosity control via controlled hydration or moderate operating temperatures, (ii) selection of GRAS-grade, biocompatible components, (iii) polarity matching to target metabolites, (iv) thermal stability of both solvent and bioactives, (v) feasibility of solvent recovery through dilution or precipitation, and (vi) compatibility with process-intensification techniques such as ultrasound or microwave irradiation [153,165,166].

### 4.4. Biocompatibility, Biodegradability and Toxicity Considerations

Although NADES are widely regarded as green solvents due to their natural origin, their biocompatibility and environmental impact are strongly composition-dependent [159,160]. Many NADES formulated from sugars, organic acids, and amino acids exhibit low toxicity and high biodegradability [167], often exceeding 90% within 28 days, and can enhance the stabilization of sensitive bioactives compared to conventional ionic liquids. However, although many NADES components are individually recognized as safe, the formation of eutectic systems results in new hydrogen-bonded structures with distinct physicochemical properties. These systems may exhibit altered bioavailability, toxicity, and environmental behavior compared to their individual constituents [18].

An additional concern is the potential co-extraction of trace elements during NADES-based extraction [168]. Recent studies have shown that most NADES systems exhibit low recovery of trace metals such as Ba, Ca, Fe, Mg, Mn, Sr, and Zn, while toxic elements such as As and Co were not detected above quantification limits. Importantly, risk assessment indicators, including hazard quotient, hazard index, and carcinogenic risk, were found to be below critical thresholds, suggesting minimal health risk under tested conditions [168,169]. However, the recovery of elements is influenced by NADES composition and water content, highlighting the need for system-specific evaluation [169]. Similarly, studies on acid-based NADES have demonstrated that hydrogen-bond donor composition plays a key role in the co-extraction of elements. Although correlations between metabolite recovery and metal content have been reported, calculated exposure metrics indicate that such extracts remain within safe limits for both ingestion and topical application [170]. These findings support the potential safety of NADES systems but emphasize the importance of composition-dependent risk assessment.

Accordingly, systematic toxicity assessment, life cycle analysis, and careful component selection are essential steps. For microalgal biorefineries, optimal NADES formulations should prioritize GRAS-grade constituents, biodegradability, low environmental impact, and minimal interference with downstream purification processes [159,160,171]. These observations further emphasize the need for composition-specific evaluation of NADES systems when applied to bioactive extraction.

### 4.5. Regulatory and Safety Considerations of NADES-Based Extraction Systems

Natural deep eutectic solvents (NADES) are increasingly considered as sustainable alternatives to conventional organic solvents; however, their regulatory status and safety evaluation remain important considerations for industrial application [154]. Although many NADES components, such as choline chloride, organic acids, sugars, and amino acids, are individually recognized as safe (GRAS), the formation of eutectic systems results in new physicochemical environments that cannot be evaluated solely based on the properties of their individual constituents [161].

One of the primary regulatory concerns relates to residual solvent presence in final products. For applications in food, nutraceuticals, and cosmetics, regulatory authorities require strict control of solvent residues. While conventional solvent limits are defined in guidelines such as ICH Q3C for residual solvents, equivalent standardized limits for NADES are not yet established, and acceptable levels are typically assessed on a case-by-case basis depending on composition and intended use [172,173].

Another critical aspect is the toxicity assessment of NADES systems. Although their individual components are often non-toxic, the hydrogen-bonded network formed in NADES can significantly alter physicochemical properties, including viscosity, polarity, and biological interactions. Studies have shown that NADES toxicity is highly composition-dependent, with some systems exhibiting cytotoxic or inhibitory effects depending on pH, concentration, and component interactions [172,174]. Therefore, safety evaluation should be conducted at the system level, rather than inferred from individual components.

From a regulatory perspective, the absence of harmonized guidelines for NADES represents a key barrier to commercialization. NADES-based extracts may fall under different regulatory categories, including food ingredients, processing aids, or novel substances, depending on their application [162]. In regions such as the European Union and the United States, approval pathways may require comprehensive toxicological assessment, stability data, and evaluation of residual solvent levels before market authorization [175].

In addition, process-related considerations, such as solvent recovery, recyclability, and scalability, are closely linked to regulatory acceptance. Efficient removal or reuse of NADES is necessary to ensure product purity and compliance with safety requirements. However, recovery efficiency can be limited by high viscosity and strong solute–solvent interactions, which remain challenges for industrial implementation [161,172].

Overall, while NADES offer advantages in terms of sustainability and extraction efficiency, their regulatory acceptance depends on systematic evaluation of safety, residue levels, and process compatibility. Future research should focus on standardized toxicity assessment, development of regulatory frameworks, and validation of NADES-based extraction systems under industrial conditions.

## 5. Integration of Process-Intensification Technologies with NADES for Microalgal Bioactive Extraction

The integration of Natural Deep Eutectic Solvents (NADES) with process-intensification technologies represents a highly promising strategy for the efficient and sustainable recovery of microalgal bioactive compounds. NADES can partially disrupt microalgal cell walls and enhance access to intracellular metabolites of microalgae. Their low toxicity, biodegradability, and tunable solvation properties render them well-suited for green biorefinery applications [153,176]. For example, choline chloride-based NADES have enabled the selective recovery of eicosapentaenoic acid (EPA) from *Nannochloropsis gaditana* [177], whereas choline chloride–acetic acid (1:2) systems have demonstrated effective extraction of proteins and carotenoids from *Neochloris texensis* and *Scenedesmus protuberans* [18,171].

Similarly, choline chloride-1,2-butanediol (1:4) NADES, supplemented with water, have been optimized for the extraction of carotenoids and phenolic compounds from *Chlorella vulgaris*, illustrating the capacity of NADES to be tailored for the selective recovery of diverse metabolite classes [178]. Collectively, these studies demonstrate that NADES composition can be rationally designed to target proteins, phenolics, pigments, omega-3 fatty acids, and lipids with substantially reduced environmental and toxicological impacts compared with conventional organic solvents [18,158,179].

**Figure 5:**
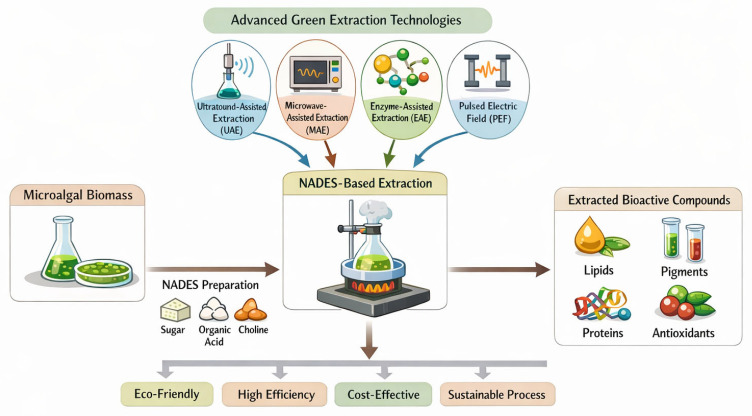
Overview of integrated natural deep eutectic solvent (NADES)-based extraction approach with advanced green extraction strategies of microalgal biomass.

The performance of NADES is further enhanced when coupled with process-intensification technologies such as ultrasound-assisted extraction (UAE), microwave-assisted extraction (MAE), enzyme-assisted extraction (EAE), pressurized liquid extraction (PLE/ASE), and supercritical CO_2_ extraction (SFE). These hybrid approaches markedly improve mass transfer, promote cell wall disruption, and increase extraction yields. Water-rich NADES have been successfully integrated with UAE and MAE for the recovery of a wide range of bioactives, while combinations such as UAE-EAE, MAE-PLE, UAE-MAE, and EAE-SFE enable multi-component fractionation within a single workflow (Figure 5) [17]. Such hybrid configurations are particularly advantageous for microalgal biomass, which contain complex mixtures of lipids, proteins, carbohydrates, and carotenoids relevant to food, pharmaceuticals, and cosmetics.

Recent studies have reported the efficient extraction of astaxanthin from *Haematococcus pluvialis* using hydrophobic NADES composed of oleic acid paired with thymol, menthol, or geraniol [180]. Among these systems, thymol–oleic acid (3:1) achieved extraction efficiencies of up to 83%, outperforming menthol- and geraniol-based formulations and oleic acid alone. This enhanced performance is attributed to the favorable physicochemical properties, including reduced viscosity, high solute affinity, and microstructural compatibility between the ester-rich NADES and carotenoid molecules [180].

From an industrial perspective, NADES-process intensification coupling offers multiple advantages, including reduced extraction time, lower energy demand, minimal solvent consumption, and a smaller environmental footprint [3]. Optimal performance requires careful selection of the solvent composition and extraction technology, which is informed by the physicochemical properties of the target metabolites and the structural complexity of the microalgal cell wall [181]. Overall, the integration of NADES with advanced extraction technologies provides a versatile and environmentally sustainable pathway for valorizing microalgal biomass, laying the foundation for next-generation low-waste biorefinery frameworks [182] (Table 4). The subsequent sections examine specific hybrid configurations—namely, NADES combined with UAE, MAE, EAE, SFE, and ASE—and discuss their mechanistic advantages and industrial potential. NADES recovery and reuse remain critical for industrial implementation. Recovery strategies include anti-solvent addition, membrane separation, and back-extraction techniques. However, recovery efficiency is influenced by viscosity, solute–solvent interactions, and system polarity, and large-scale application remains a challenge [182].

### 5.1. Ultrasound-Assisted Extraction Coupled with NADES (UAE-NADES)

UAE is one of the most widely applied techniques for microalgal bioprocessing owing to its reduced solvent demand, enhanced mass transfer, and short extraction times. UAE operates through acoustic cavitation, whereby ultrasonic waves (typically 20–100 kHz) generate and collapse microbubbles, producing localized hotspots characterized by transient high pressures and temperatures. The resulting microjets and shear forces disrupt algal cell walls, facilitate solvent penetration, and lower diffusion barriers, enabling the rapid release of intracellular metabolites [121,196,197].

UAE performance is governed by several interdependent parameters, including ultrasonic frequency, power input, temperature, and solvent properties. Frequencies in the range of 20–30 kHz promote intense cavitation, whereas excessive power can induce bubble coalescence, local overheating, and degradation of thermolabile compounds [198,199]. The solvent viscosity, vapor pressure, and surface tension further influence cavitation efficiency, with low-viscosity systems favoring bubble dynamics and mass transfer. Importantly, UAE enables the processing of wet biomass, substantially reducing the energy demand associated with biomass drying. Despite these advantages, challenges remain, particularly related to scale-up, non-uniform acoustic field distribution, probe erosion, and the need to maintain mild conditions to preserve bioactive compounds. These limitations have driven interest in coupling UAE with green solvents, such as NADES [137].

The integration of UAE with NADES has emerged as a highly effective strategy for the extraction of microalgal compounds. NADES, formed from natural hydrogen bond donors and acceptors, offer tunable polarity, low volatility, strong solvation capacity, and high biocompatibility. When combined with cavitation-induced cell disruption, NADES-UAE systems markedly enhance mass transfer and extraction efficiency while minimizing reliance on hazardous organic solvents [180,185]. This synergy couples the mechanical effects of ultrasound with the benign and selective solvation properties of the NADES.

Recent studies have highlighted the versatility of UAE-NADES systems for multiple metabolite classes. Martins et al. [123] applied a choline chloride-sugar NADES (1:2) to *Spirulina platensis*, achieving pigment yields of approximately 165 mg g^−1^ dry biomass under optimized conditions (60 °C, 20 min, solvent-to-biomass ratio 70 mL mg^−1^), alongside notable antioxidant activity. Building on this approach, Zin et al. [183] developed a DES-based UAE protocol for *Spirulina*, achieving protein recovery exceeding 80% with high protein concentration and strong antioxidant, emulsifying, and foaming properties, and selective cytotoxicity against colorectal cancer cell lines highlighting its suitability for food and nutraceutical applications.

Similarly, UAE-NADES systems have been successfully applied to *Chlorella vulgaris*, where a choline chloride-urea formulation enabled efficient protein recovery with high digestibility, functional performance, and selective cytotoxicity against colorectal cancer cell lines, while exhibiting low toxicity toward normal cells [184]. In addition to proteins and pigments, UAE-NADES facilitates the efficient extraction of carotenoids and lipids. For example, canthaxanthin was recovered from *Chromochloris zofingiensis* using an octanoic acid–decanoic acid hydrophobic deep eutectic solvent (DES), achieving 70.4 µg mL^−1^ canthaxanthin under optimized conditions (50 °C, 49 min, molar ratio 2.3:1, LSR 66.2 mL g^−1^). This high yield was attributed to the synergistic effects of DES-induced cell erosion and ultrasonic cavitation [200]. In another study, brief NADES-UAE using choline chloride and acetic acid pretreatment enhanced lipid extraction from *Chlorella pyrenoidosa* [186]. The best optimized condition of methanol-to-n-butanol ratio of 1:4, a solvent-to-biomass ratio of 30:1 (*v*/*w*), and extraction at 60 °C for 120 min, yielding biodiesel-grade fatty acid profiles including palmitic (C16:0), linoleic (C18:2), and linolenic (C18:3) acids, compliant with international fuel standards [186].

Collectively, UAE-NADES systems demonstrate substantial potential for the selective, low-solvent extraction of high-value microalgal compounds. Key considerations for optimal performance include the viscosity reduction of NADES, commonly achieved through controlled water addition (20–40 wt%), the selection of food-grade formulations for nutraceutical applications, moderate operating temperatures to protect thermolabile metabolites, and ultrasonic reactor designs capable of delivering sufficient power density for viscous eutectic matrices. Despite these advantages, limitations remain, including variability in extraction efficiency, challenges in solvent recovery, and limited validation at pilot or industrial scale.

### 5.2. Microwave-Assisted Extraction Coupled with NADES (MAE-NADES)

Microwave-assisted extraction (MAE) operates through dielectric heating, primarily driven by dipole rotation and ionic conduction when microwave radiation (typically 915 MHz or 2.45 GHz) interacts with polar solvents and intracellular water molecules. Unlike conventional conductive heating, microwave energy is deposited volumetrically, enabling rapid and uniform elevation of the temperature. This heating mode promotes efficient disruption of microalgal cell walls, accelerates solute diffusion, and enhances extraction kinetics, particularly for species with rigid or multilayered cell-wall structures [201,202,203]. MAE has been effectively applied to recover pigments, PUFA-rich lipids, antioxidants, and proteins with reduced solvent consumption and shortened processing times [204].

The extraction efficiency of MAE is governed by multiple parameters, including microwave power, irradiation time, solvent polarity, temperature, and biomass loading. Polar solvents with high dielectric losses efficiently absorb microwave energy, whereas mixed solvent systems can balance microwave absorption with solute solubility. However, prolonged irradiation increases the risk of pigment bleaching, lipid oxidation, and degradation of thermolabile compounds, necessitating careful optimization [205]. Additional challenges, such as non-uniform electromagnetic field distribution, limited penetration depth, high equipment cost, and scale-up constraints, currently restrict large-scale implementation. Despite these limitations, MAE remains attractive for integrated biorefinery operations because of its high efficiency, short processing times, and compatibility with green solvent systems, including NADES [206,207].

The coupling of MAE with NADES has emerged as a promising strategy for sustainable microalgal bioprocessing. NADES offer tunable polarity, low volatility, and strong solvation capacity, and when combined with volumetric microwave heating, they enable efficient cell disruption and enhanced mass transfer, even within viscous solvent matrices. Because many NADES exhibit relatively high viscosity and lower dielectric loss than water or ethanol, partial hydration—typically through the addition of 20–40 wt% water, is often required to improve the microwave absorption and extraction performance [188].

Several studies have illustrated the advantages of MAE-NADES systems for diverse metabolite classes. Tommasi et al. [15] evaluated multiple choline chloride-based DES paired with oxalic acid, levulinic acid, urea, ethylene glycol, or sorbitol for lipid extraction from *Phaeodactylum tricornutum*. Carboxylic-acid-based DES demonstrated superior performance, achieving total fatty acid recoveries of up to 80% and significantly enhanced selectivity (88%) compared with conventional Bligh–Dyer extraction (35%). Moreover, DES-microwave pretreatment markedly improved subsequent supercritical CO_2_ extraction, increasing lipid recovery by up to twenty-fold and yielding a triglyceride-rich fraction.

MAE-assisted green solvent systems also show strong potential for protein recovery. Motlagh et al. [187] applied an ionic-liquid-assisted MAE process using choline acetate for protein extraction from *Nannochloropsis oceanica*, achieving protein yields of 26.35% equivalent to 65.06% of total extractable protein under optimal conditions (0.5 g biomass, 2% *w*/*v* [Ch] [Ac], 40 °C, 30 min). substantially higher than those obtained via Soxhlet extraction (0.63% yield under optimized conditions). Similarly, Zin et al. [188] employed a choline chloride-urea DES in MAE to extract proteins from *Spirulina* and *Chlorella*, under optimized microwave conditions (160 W; biomass-to-solvent ratio of 1:40 for *Spirulina* and 1:30 for *Chlorella*; 10 min), protein yields of 30.48% and 15.53% were obtained, with balanced amino acid profiles. The extracted proteins exhibited strong antioxidant activity, selective cytotoxicity toward colorectal cancer cell lines, and favorable functional properties, including high foaming and emulsifying capacities.

Beyond proteins, MAE-NADES systems have proven to be effective for extracting phenolic compounds and other bioactives. Fihri et al. [189] demonstrated that choline chloride–citric acid DES outperformed conventional solvents in recovering phenolics 4.98 mg/g from *Chlorella vulgaris* and *Scenedesmus incrassatulus*, yielding higher antioxidant activity and notable antibacterial effects against *Staphylococcus aureus*. These findings highlight the versatility of citric-acid-based DES as sustainable solvents for the recovery of microalgal bioactives.

Collectively, these studies underscore the effectiveness of MAE–NADES as a rapid, energy-efficient, and environmentally favorable extraction strategy. When appropriately optimized, MAE-NADES systems enable the high-yield recovery of functional proteins, lipids, and phenolic compounds, supporting their growing potential in nutraceutical and pharmaceutical applications and reinforcing their role in next-generation sustainable microalgal biorefineries. Despite these advantages, limitations remain, including variability in extraction efficiency, challenges in solvent recovery, and limited validation at pilot or industrial scale.

### 5.3. Enzyme-Assisted Extraction Coupled with NADES (EAE-NADES)

Enzyme-assisted extraction (EAE) exploits hydrolytic enzymes to selectively degrade structural polysaccharides and glycoproteins in microalgal cell walls, enabling the release of intracellular metabolites under mild-processing conditions. This approach minimizes thermal degradation, reduces solvent dependence, and enhances selectivity, making it particularly attractive for recovering sensitive bioactives [208,209]. Enzyme selection is dictated by cell wall composition: cellulose- and hemicellulose-rich species respond effectively to cellulases and hemicellulases, whereas peptidoglycan-containing taxa, such as *Arthrospira* and *Nostoc*, are more susceptible to lysozymes or proteases [210,211,212,213,214].

EAE typically operates at moderate temperatures (30–55 °C) and near-neutral pH (5–7), following Michaelis–Menten kinetics, which are governed by enzyme dosage, substrate accessibility, and reaction time. When combined with auxiliary techniques, such as ultrasound or green solvent systems, EAE benefits from enhanced mass transfer and improved extraction efficiency. Importantly, enzymatic pretreatment preserves the structural integrity of pigments, polyunsaturated fatty acids, phycobiliproteins, and phenolic compounds, while also enabling the generation of value-added derivatives, such as antioxidant peptides and oligosaccharides [210,211,212,213,214]. Despite these advantages, their widespread implementation is constrained by enzyme cost, extended reaction times, product inhibition, and enzyme sensitivity to solvent composition, ionic strength, and extreme pH. Strategies such as enzyme immobilization and recycling have been explored to improve economic viability [215,216].

The integration of EAE with NADES represents an emerging and highly promising strategy for selective microalgal bioprocessing within a green extraction framework. In suitably designed NADES, hydrogen-bonding networks can stabilize the enzyme conformation by mimicking natural hydration shells, potentially enhancing catalytic activity and operational stability [217]. However, careful solvent design is essential because high viscosity or ionic strength may impede substrate diffusion or compromise enzyme performance. Enzymatic pretreatment weakens rigid cell wall matrices composed of cellulose, hemicellulose, alginate, and glycoproteins, increasing porosity and facilitating access to intracellular metabolites. Therefore, the optimization of enzyme composition, concentration, pH, temperature, biomass loading, and hydrolysis time is critical to maximize cell wall disruption and extraction efficiency [217].

Although the direct application of EAE-NADES in microalgal systems remains limited, insights from related biomass streams are informative. Vo et al. [195] demonstrated the effectiveness of a combined UAE-EAE-NADES approach for extracting tannins, flavonoids, and terpenoids from spent tea leaves, with acetic acid–glycerol and acetic acid–glucose NADES showing high selectivity for different metabolite classes. Simultaneous enzymatic and ultrasonic treatments in the presence of NADES yielded the highest recoveries and bioactivities, with scanning electron microscopy confirming extensive structural disruption [195]. Similar benefits of enzymatic pretreatment have been reported when integrated with UAE, MAE, pressurized liquid extraction, and supercritical CO_2_ extraction for enhanced recovery of phenolics and other bioactives [19,218].

Overall, although EAE has been widely validated for phytochemical recovery from plant and algal biomass, its systematic integration with NADES for microalgal extraction remains underexplored. Evidence from plant-based systems suggests that coupling enzymatic pretreatment with NADES could form a next-generation sustainable extraction platform, offering improved yields and selectivity, reduced processing time and energy demand, and enhanced environmental performance within microalgal biorefinery frameworks. Despite these advantages, limitations remain, including variability in extraction efficiency, challenges in solvent recovery, and limited validation at pilot or industrial scale.

### 5.4. SCF/SCCO_2_-NADES Extraction

Supercritical fluid extraction (SFE) employs solvents above their critical temperature and pressure, with carbon dioxide (CO_2_) being the most widely used owing to its moderate critical point (31.1 °C, 7.38 MPa), non-toxicity, and facile removal from extracts. Under supercritical conditions, CO_2_ behaves as a single homogeneous phase exhibiting gas-like diffusivity and liquid-like solvating power, enabling the efficient penetration of microalgal matrices and extraction of non-polar to moderately polar compounds such as lipids, sterols, and carotenoids [219,220]. The solvation strength increases with pressure through density enhancement, whereas the temperature exerts a dual influence by elevating the solute vapor pressure and potentially reducing the solvent density. The addition of polar co-solvents, such as ethanol, further expands the polarity window, facilitating the recovery of a broader range of metabolites from the sample. Key operational parameters, including the CO_2_ flow rate, particle size, and biomass moisture content, strongly influence the extraction efficiency, with low residual moisture typically preferred. SFE is particularly attractive for food and pharmaceutical applications because it yields solvent-free extracts with preserved bioactivity and minimal oxidative degradation [221]. However, high capital costs, operational complexity, limited efficacy for polar compounds, and scale-up challenges often restrict its standalone use, positioning SFE as a complementary component within integrated biorefinery schemes [222,223].

The integration of Natural Deep Eutectic Solvents (NADES) with supercritical CO_2_ (SC-CO_2_) represents an advanced strategy for broadening solvent polarity, enhancing mass transfer, and improving sustainability in bioactive recovery. NADES can function as pretreatment media that weaken microalgal cell walls, thereby facilitating access to intracellular metabolites during subsequent SC-CO_2_ extraction. In some configurations, small amounts of NADES, particularly those that are partially volatile or CO_2_-dispersible, may also act as polarity modifiers, enabling the extraction of moderately polar compounds without the use of petroleum-derived co-solvents. This complementary interaction combines the solvent-free advantages of SC-CO_2_ with the tunable solvation and cell disruption capabilities of NADES, reducing the dependence on hazardous organic solvents and improving environmental performance.

The integration of NADES with supercritical CO_2_ (SCCO_2_) is an advanced strategy. This can broaden solvent polarity, improve mass transfer, and improve sustainability in microalgal bio-product recovery. NADES can be used as a pretreatment medium which weakens the microalgal cell wall and provides a gateway for the access to intracellular metabolites for CO_2_ extraction. Sometimes, trace quantities of NADES, if partially volatile or CO_2_-dispersible, may also function as modifiers, and enhance the polarity of SCCO_2_. This also helps in the extraction of moderately polar compounds without resorting to petroleum-based co-solvents [224]. While SCCO_2_ enhances the extraction of non-polar metabolites, the modulation of NADES facilitates the extraction of a broader range of compounds across the polarity spectrum. This is particularly beneficial by reducing dependency on the harmful organic solvents, which ultimately improve the environmental credentials of the process [13,224]. This is a very productive and sustainable approach for obtaining high value metabolites from microalgae, which are critical for the pharmaceutical, food, cosmetics, and nutraceutical industries [224].

Although the application of SC-CO_2_-NADES in microalgal systems remains limited, studies on plant matrices have illustrated the potential of this hybrid approach. For example, the coupling of SC-CO_2_ with menthol–lactic acid NADES substantially enhanced curcuminoid recovery from turmeric, yielding extraction efficiencies that are orders of magnitude higher than those achieved with SC-CO_2_ alone, ultrasound-assisted NADES extraction, or conventional ethanol-based methods [225]. The resulting extracts exhibited strong antioxidant activity and inhibitory effects against key enzymes, highlighting the functional advantages of hybrid processing [225]. Similarly, the integration of ultrasound pretreatment, SC-CO_2_ extraction, and NADES fractionation enabled the selective recovery of terpenes and polyphenols from *Lavandula stoechas* [226]. After the SCCO_2_ extraction of oxygenated monoterpenes, the remaining biomass was treated with NADES composed of betaine–ethylene glycol (Bet:EG), betaine–glycerol (Bet:Gly), and glycerol–glucose (Gly:Glu) at 30 and 60 °C. The highest yield was recorded from Bet:EG solvent at 60 °C, dominated by rutin (438.93 µg/mL). The process produced extracts rich in bioactive flavonoids, with notable antioxidant and antibacterial properties [226].

Collectively, these studies demonstrate that SC-CO_2_-NADES coupling offers a powerful platform that unites high-purity, solvent-free supercritical extraction with the selectivity and versatility of green eutectic solvents. Although validation in microalgal biomass remains in its early stages, plant-based evidence provides a valuable roadmap for adaptation to microalgal systems, where rigid cell walls and heterogeneous metabolite distributions present additional challenges for analysis. Future development may benefit from the integration of emerging process-intensification tools, such as pulsed electric fields, ohmic heating, centrifugal partition extraction, and surfactant-mediated extraction, to enhance efficiency and scalability [21,158,227]. Overall, SC-CO_2_-NADES systems represent a promising next-generation approach for the selective, sustainable, and high-value extraction of microalgal bioactive compounds, warranting continued investigation and optimization for industrial deployment.

### 5.5. Accelerated Solvent Extraction Coupled with NADES (ASE-NADES)

Accelerated solvent extraction (ASE), also referred to as pressurized liquid extraction (PLE) or subcritical water extraction (SWE) when water is employed, has emerged as an efficient and environmentally benign technology for recovering bioactive compounds under elevated temperatures and pressures [228]. ASE operates by applying temperatures typically between 50 and 250 °C and pressures of 10–20 MPa to enhance solvent strength while maintaining the liquid phase [229]. An increase in temperature reduces the solvent viscosity and increases the diffusivity, thereby improving the mass transfer. In the case of water, the dielectric constant decreases markedly from approximately 80 at 25 °C to ~27 at 250 °C, enabling solubilization of moderately non-polar metabolites such as phenolics, carotenoids, and selected lipids. This temperature-dependent tunability supports selective extraction across polarity gradients and facilitates multiproduct biorefinery strategies that are more sustainable [230,231].

SWE is particularly effective for microalgal biomass, as pressurized water readily penetrates complex cell wall architectures without requiring harsh pretreatments. At lower temperatures, highly polar compounds such as carbohydrates and amino acids are preferentially extracted, whereas higher temperatures favor the recovery of pigments and fatty acids [232]. Optimal performance requires careful balancing of temperature, compound stability, and solvent-to-solid ratios, typically in the range of 10:1–30:1, to maximize extraction efficiency while avoiding excessive dilution. Pressurized Liquid Extraction (PLE) extends these principles to alternative green solvents, including ethanol and NADES, broadening the accessible polarity range and enabling the enhanced recovery of carotenoids and polyphenols [233,234]. Nonetheless, ASE-based approaches face challenges related to high-pressure equipment requirements, elevated energy demand at scale, potential degradation of thermolabile compounds, and increased process complexity [235].

The integration of NADES into ASE workflows offers a promising route to expand solvent selectivity and preserve temperature-sensitive metabolites under moderate subcritical conditions. When appropriately designed, NADES can function as effective subcritical solvents, enabling high recovery with reduced extraction times and solvent usage. However, their intrinsic viscosity, thermal stability under elevated temperatures and pressures, and solvent recovery are critical considerations. Therefore, successful industrial implementation will depend on optimizing the NADES composition to reduce viscosity under operating conditions, ensuring compatibility with high-pressure systems, and developing efficient recycling strategies [3].

Recent studies underscore the potential of NADES in intensified extraction schemes; for example, Zhou et al. [16] reported that ultrasonic enzyme-assisted DES extraction of polysaccharides achieved yields more than threefold higher than conventional hot-water extraction, producing lower molecular weight fractions with enhanced antioxidant activity. Density functional theory analysis indicated stronger hydrogen-bond interactions between the DES and glucan structures than between the DES and water, rationalizing the improved solubility and extraction efficiency [16]. Complementary work on the alkaline subcritical water extraction of *Ascophyllum nodosum* demonstrated the rapid recovery of antioxidant-rich fractions. The highest yields of crude extract and alginate were achieved at 160 °C, 18 min, and 0.1 g mass loading. An increase in temperature up to 200 °C increased the phenolic content and antioxidant activity; however, it also resulted in the partial degradation of polysaccharides such as alginate and fucoidan, with extraction kinetics well described by Fickian diffusion. However, excessive temperatures promoted the partial degradation of polysaccharides, highlighting the need for precise process control [14].

Overall, ASE and SWE represent robust green extraction platforms, and their coupling with NADES offers promising pathways for the selective, scalable, and low-impact processing of microalgal biomass. To fully realize the potential of ASE-NADES systems in next-generation microalgal biorefineries, further research is required to address solvent stability, viscosity management, and comprehensive life-cycle performance. Despite these advantages, limitations remain, including variability in extraction efficiency, challenges in solvent recovery, and limited validation at pilot or industrial scale.

### 5.6. Energy Consumption of MAE, UAE and Enzymatic Hydrolysis

Pretreatment technologies, such as microwave-assisted extraction (MAE), ultrasound-assisted extraction (UAE), and enzymatic hydrolysis, can substantially enhance microalgal extraction efficiency but are also associated with distinct energy demands and environmental footprints. Therefore, a comparative assessment of these processes is essential to guide the selection of energy-efficient strategies for industrial deployment. Energy consumption varies significantly among extraction techniques, with conventional methods requiring high thermal input, whereas ultrasound- and microwave-assisted systems reduce processing time and energy demand by up to 30–60% under optimized conditions [236,237].

Zhao et al. [193] evaluated the energy consumption of MAE and UAE during essential oil extraction from *Cuminum cyminum* seeds using a choline chloride–lactic acid (1:3) NADES. Their analysis indicated higher energy consumption for UAE than for MAE, corresponding to a 24.24% increase in CO_2_ emissions. However, other studies have reported comparable energy requirements for MAE and UAE, underscoring the strong influence of solvent composition, biomass characteristics, and equipment configuration on overall energy profiles [193].

Santana et al. [238] employed ternary NADES composed of citric acid, malic acid, xylitol, and water for trace metal extraction from animal and plant fibers, demonstrating high extraction efficiencies (80–120%) using both MAE and UAE. Sustainability was assessed using the Analytical Eco-Scale, where scores above 75 indicated eco-friendly processes. NADES-based extraction achieved scores of 97 for UAE and 96 for MAE, with differences primarily attributed to energy demand (<0.1 kW for UAE-treated samples versus <1.5 kW for MAE-treated samples). These findings contrast with those of Zhao et al. [193], reinforcing that energy consumption cannot be generalized across extraction platforms and is highly system-dependent.

Enzymatic hydrolysis represents a comparatively low-energy alternative, as the energy input is largely confined to moderate-temperature incubation and mechanical mixing. However, extended residence times and strict control of operational parameters, including pH, temperature, and aeration, can increase overall processing costs, despite the low instantaneous energy demand. Collectively, these observations highlight that pretreatment energy consumption is governed by multiple interacting factors, including solvent properties, biomass composition, reactor design, and process scale. Systematic side-by-side evaluations of MAE, UAE, and enzymatic hydrolysis under standardized conditions are still required to accurately define energy footprints and operational expenditures. Such analyses are critical for identifying the most energy-efficient and scalable pretreatment strategies for next-generation microalgal biorefineries for biofuel production [217].

## 6. Sustainability Assessment and Techno-Economic Considerations

The economic viability and environmental performance of extraction technologies are critical determinants of industrial adoption. For the NADES-assisted green extraction of microalgal bioactives, sustainability and commercial feasibility are primarily evaluated through integrated environmental and economic analyses. Life-cycle assessment (LCA) quantifies cradle-to-gate environmental impacts, including energy demand, emissions, and resource use, whereas techno-economic analysis (TEA) assesses capital investment, operating costs, process throughput, and sensitivity to key operational parameters of a process. This section synthesizes the current LCA insights for NADES-based extraction systems, outlines a TEA framework tailored to microalgal biorefineries, and discusses the regulatory, safety, and environmental considerations governing applications in the food, pharmaceutical, and nutraceutical sectors.

### 6.1. Life-Cycle Assessment (LCA): Frameworks and NADES vs. Conventional Solvents

Life cycle assessment (LCA) provides a standardized framework, as defined by ISO 14040/44, for quantifying the environmental impacts associated with solvent-based extraction processes, including global warming potential, cumulative energy demand, eutrophication, and toxicity [239,240]. In extraction systems, the principal contributors to life cycle impacts include solvent precursor production, energy consumption during extraction operations such as heating, pressurization, and mixing, solvent losses during downstream processing, and end-of-life emissions or biodegradation [241,242]. Although the LCA literature on NADES remains limited, existing studies indicate that their environmental performance is highly context-dependent. NADES typically reduce environmental burdens when their constituents are bio-based or low-impact co-products, solvent formulations require minimal energy input, and recovery and reuse efficiencies are high, commonly exceeding five to ten cycles. Conversely, when NADES are synthesized from energy-intensive or petrochemical feedstocks or when solvent recovery is inefficient, cradle-to-gate impacts may exceed those of water, ethanol, and conventional organic solvents. Recent life cycle assessments (LCAs) have concluded that the sustainability of NADES-assisted extraction is governed primarily by solvent composition, precursor sourcing, and recycling efficiency [21,239,240].

In the context of microalgae, the evaluation of NADES-based extraction processes must consider specific characteristics of the biomass, including cell wall rigidity, water content, and biochemical composition. These factors directly influence extraction efficiency, energy demand, and solvent consumption [243]. Compared to terrestrial biomass, microalgae often require additional pretreatment steps, which can increase process complexity and cost [243]. Therefore, techno-economic evaluation should incorporate parameters such as biomass productivity, extraction yield, solvent recyclability, and energy input to provide a realistic assessment of process feasibility.

Representative case studies illustrate these dependencies. Alanazi et al. [239] demonstrated environmental advantages for NADES-microwave-assisted cellulose extraction from date-palm residues, contingent on high solvent recycling rates and low-impact constituent sourcing. Similar LCA evaluations of NADES-based flavonoid and cellulose extraction report reduced toxicity and volatile organic compound emissions but show that these benefits diminish when purification energy demands or feedstock impacts are substantial [239]. For microalgal biorefineries, three methodological considerations are particularly critical: inclusion of solvent manufacturing within system boundaries, explicit modeling of solvent recycling scenarios, and normalization of impacts to product yield. Environmental benefits scale strongly with solvent reuse, and comparisons across no-recovery, moderate-recycling, and high-recycling scenarios are essential [244,245]. Moreover, yield normalization is crucial, as higher extraction efficiencies can substantially reduce impacts per kilogram of product even when energy input per unit biomass is elevated.

### 6.2. Techno-Economic Analysis (TEA): Parameters and Example Workflow

Techno-economic analysis (TEA) complements the LCA by quantifying financial feasibility. In NADES-assisted microalgal extraction technologies, key economic variables include the cost of solvent constituents and formulation, energy demand during solvent preparation, solvent recovery and recycling costs, extraction energy requirements, throughput and residence time, capital expenditure for specialized equipment, product yield and purity, and operating costs related to labor, utilities, maintenance and waste treatment [246]. Standard TEA workflows are built on mass- and energy-balanced process flowsheets, followed by equipment sizing and cost estimations. Unit production costs are typically derived by annualizing operating expenses and depreciation, with sensitivity analyses identifying the solvent price, recycling rate, extraction yield, and product value as dominant economic drivers. Comparative TEA scenarios contrasting conventional solvent extraction with low- and high-recycling NADES systems are particularly informative, as they define the break-even recycling thresholds and cost ceilings for the components of NADES required for commercial viability [246].

### 6.3. Regulatory and Market-Readiness Costs

Regulatory and market-readiness costs represent additional determinants of feasibility, particularly for food and pharmaceutical applications. Regulatory frameworks prioritize residual solvent control and safety of both solvent constituents and final extracts. While many NADES components are generally recognized as safe, regulatory approval requires validated analytical data demonstrating acceptable residual levels or confirming that residual constituents are approved ingredients [247,248]. Regulatory dossiers typically include analytical method validation, toxicological assessments where necessary, and allergenicity or safety evaluations. These compliance costs, which may range from tens to hundreds of thousands of USD depending on jurisdiction and application, must be incorporated into TEA models [249,250].

From a regulatory perspective, the application of NADES in food, pharmaceutical, and cosmetic systems remains limited by the absence of specific guidelines. Existing regulatory frameworks are primarily designed for conventional solvents and do not directly address the complexity of eutectic systems [162]. As a result, NADES-based products are typically evaluated on a case-by-case basis, requiring detailed information on composition, residual levels, and toxicological profiles. The lack of standardized testing protocols and regulatory thresholds for NADES residues represents a key barrier to commercialization and highlights the need for coordinated efforts to establish clear evaluation criteria [162,173].

### 6.4. Safety and Toxicity Comparison

From a safety and toxicity perspective, NADES offer clear advantages over conventional organic solvents because of their low volatility, reduced toxicity, biodegradability, and lower occupational risks. In contrast, traditional solvents such as methanol and hexane present significant health, flammability, and environmental hazards issues [21,227,251,252]. However, the high viscosity of many NADES remains a central practical limitation, as it can restrict mass transfer, complicate solvent recovery, and hinder scaling-up [3,13,21,253]. Viscosity can be mitigated through moderate heating, controlled water addition, or co-solvent incorporation, where permissible, although excessive dilution may disrupt the hydrogen-bond networks critical for solvation. When effectively managed and recycled, NADES systems can substantially enhance sustainability and economic performance [13,253].

Toxicity assessment of NADES systems remains an area of active research, and available studies indicate that their biological effects are highly dependent on composition and concentration [13,253]. While some NADES formulations have shown low cytotoxicity in vitro models, others have demonstrated inhibitory effects on microbial growth and cellular activity, particularly at higher concentrations or under specific pH conditions. Importantly, these effects cannot be predicted solely based on the safety of individual components, as the hydrogen-bonded structure of NADES can alter transport properties and biological interactions [167]. This highlights the need for systematic toxicity evaluation at the system level, particularly for applications involving direct human exposure or environmental release.

### 6.5. Environmental Impact

Environmental considerations remain central to the extraction process selection. NADES-assisted techniques exhibit favorable environmental profiles owing to their negligible vapor pressure, elimination of volatile organic compound emissions, and mild synthesis conditions that reduce energy and carbon footprints relative to conventional solvent production [149,254]. In contrast, traditional solvents often require energy-intensive manufacturing, strict disposal protocols, and specialized waste treatment. For food and pharmaceutical applications, residual NADES in the final products necessitate careful analytical verification and toxicological evaluation, as eutectic interactions may alter the bioavailability of the constituents. These regulatory requirements increase capital and operating expenditures and must be explicitly addressed in feasibility analyses [246,255,256].

Overall, NADES are a compelling alternative to conventional organic solvents, offering superior safety, environmental compatibility, and extraction performance. When challenges related to viscosity management, solvent recovery, and regulatory compliance are systematically addressed, NADES-based systems provide a robust foundation for the sustainable and industrially viable recovery of microalgal bioactive compounds.

## 7. Future Perspectives and Opportunities

As microalgal biorefineries advance toward commercial implementation, NADES-based green extraction offers a transformative platform for sustainable separation, process intensification, and high-value product development. Future progress will depend on computationally guided solvent design, hybrid extraction architectures, regulatory preparedness, and coordinated research efforts that bridge laboratory discoveries with industrial deployment.

### 7.1. Task-Specific NADES Design Using Computational and AI Tools

Conventional NADES formulations have largely relied on the empirical screening of hydrogen bond donors and acceptors. Although effective at the laboratory scale, this approach is time-consuming and poorly suited to industrial requirements, where viscosity, selectivity, recyclability, and biomass-specific interactions must be optimized simultaneously [257,258,259]. Advances in computational chemistry and artificial intelligence (AI) have enabled rational, task-specific NADES design. Predictive frameworks such as COSMO-RS can estimate activity coefficients, solute–solvent interactions, and polarity, while process simulation tools (e.g., Aspen Plus) and sustainability assessment approaches, including life cycle assessment (LCA) and techno-economic analysis (TEA), support process optimization and scale-up [149,260]. In addition, machine learning models, including transformer architectures trained on SMILES datasets, have been applied to screen and identify previously unexplored eutectic systems [260].

These tools allow the pre-screening of HBA/HBD combinations for the stability, viscosity, biodegradability, and selective solvation of microalgal bioactives, such as carotenoids, polyunsaturated fatty acids, peptides, and phycobiliproteins [259]. AI-guided solvent design further offers the prospect of preferential solubilization of individual target compounds, thereby minimizing the co-extraction of chlorophylls or neutral lipids and reducing downstream purification burdens. A central challenge remains the integration of computational predictions with experimental validation in real microalgal matrices and the coupling of solvent design with extraction kinetics, biomass architecture, and transport phenomena.

### 7.2. Standardization, Demonstration Projects and Regulatory Pathways

The translation of NADES-assisted extraction from laboratory studies to industrial practice requires rigorous standardization, demonstration-scale validation, and clearly defined regulatory pathways [261,262]. The current literature remains fragmented, with inconsistent reporting of solvent composition, physicochemical properties, molar ratios, extraction conditions, recycling efficiency, and sustainability metrics, which limits cross-study comparability and hinders industrial adoption [22,149,263]. Moreover, pilot-scale data are still scarce, particularly for continuous operations at throughputs exceeding 100 kg of biomass per day with full mass and energy balance closure.

Demonstration-scale facilities are therefore essential for generating realistic performance data, evaluating solvent cycling stability, and producing integrated techno-economic (TEA) and life cycle assessment (LCA) datasets [149]. For food and pharmaceutical applications, NADES must be composed of food-grade constituents or demonstrate validated removal to acceptable residual levels. Regulatory engagement should occur early in development and include validated analytical methods for residual solvent quantification, toxicological assessment, where eutectic pairing may alter bioavailability, and process validation to ensure reproducibility [162].

Well-documented case studies integrating solvent recycling, sustainability metrics, and regulatory compliance are critical for building confidence among industrial stakeholders and regulators [246]. At present, no standardized regulatory limits have been established for residual NADES in food, pharmaceutical, or cosmetic products [162]. In contrast to conventional solvents, which are regulated under frameworks such as the ICH guidelines for residual solvents, NADES are evaluated based on their composition and intended use [248]. As a result, acceptable residual levels are typically determined on a case-by-case basis, considering factors such as toxicity, exposure route, and functional role in the final product. This lack of defined thresholds represents a key regulatory challenge for the broader application of NADES-based extraction systems [255].

### 7.3. Research Roadmap and Recommended Experimental Priorities

To accelerate implementation, a coordinated research roadmap is proposed, built on three pillars: computational solvent design, process intensification and scale-up, and integrated sustainability and economic assessments. First, task-specific NADES should be designed using COSMO-RS and AI-based tools for defined microalgal targets, followed by experimental validation of solubility, selectivity, viscosity, and mass transfer performance. Second, systematic design-of-experiment studies are required to optimize the molar ratio, water content, temperature, and solid-to-solvent ratio, with benchmarking against conventional solvents under the same conditions. Third, pilot-scale hybrid processes, such as NADES-SFE or NADES-PLE, should be evaluated under continuous operation to quantify the solvent recycling efficiency, energy demand, extraction yield, and solvent inventory, alongside protocols for solvent regeneration and multi-cycle reuse.

These technical developments must be accompanied by integrated LCA-TEA modeling to assess capital and operating costs, energy and utility requirements, solvent pricing, and recycling thresholds, supported by sensitivity analyses to identify the break-even conditions. Finally, regulatory and quality assurance studies, including residual solvent analytics, biodegradability, cytotoxicity, and allergenicity testing, should be conducted in parallel with early regulatory consultations. The convergence of computational solvent design, hybrid extraction technologies, demonstration-scale validation, and rigorous sustainability assessment provides a realistic pathway for NADES-assisted microalgal extraction to transition from a promising laboratory concept to an industrially viable platform. As solvent recovery data mature and regulatory frameworks evolve to accommodate eutectic systems, NADES may progress from niche alternatives to mainstream solvents in circular, low-waste microalgal biorefineries. The stabilizing effect of NADES on extracted bioactive compounds has also been reported, with studies demonstrating improved retention of antioxidant activity and phenolic content during long-term storage. However, similar stability studies for microalgal extracts remain limited and warrant further investigation [264,265,266].

### 7.4. Industrial and Commercial Perspective

Despite increasing research interest, the commercialization of NADES-based extraction processes for microalgal bioactives remains limited. While microalgal products such as omega-3 oils, pigments, and proteins are widely available, their production is predominantly based on conventional or supercritical fluid extraction methods. The adoption of NADES in industrial applications is still at an early stage, primarily due to challenges related to solvent recovery, regulatory approval, and process standardization. Further pilot-scale validation and regulatory clarity are required to support their industrial implementation.

## 8. Conclusions

Natural deep eutectic solvents (NADES) are a robust class of green solvents that can address persistent challenges in the extraction of microalgal bioactives. Their tunable polarity, low volatility, and biocompatibility enable the mild yet efficient recovery of pigments, lipids, phenolics, and other high-value metabolites, thereby reducing the dependence on toxic organic solvents. When integrated with process-intensification technologies such as ultrasound, microwave irradiation, pressurized liquid extraction, or supercritical CO_2_, NADES enhance mass transfer, selectivity, and overall efficiency, while lowering solvent use and simplifying downstream purification. To achieve industrial translation, NADES-assisted extraction must be supported by standardized life-cycle and techno-economic assessments, rational design of task-specific solvents, and pilot-scale validation of the extraction process. Efficient solvent recovery, regulatory alignment, and integration into circular biorefinery frameworks are critical. Overall, NADES-based platforms offer a scalable, selective, and environmentally responsible pathway for the production of high-value microalgal bioactives.

## Figures and Tables

**Table 1:** Microalgal species and their bioactive compounds, functional properties, and food applications.

Microalgal Species	Major Bioactive Compounds	Functional/Biological Properties	Food and Nutritional Applications	References
*Isochrysis galbana*	DHA (ω-3 PUFA)	Anti-inflammatory; cognition and visual support	Infant formula; yogurt; bakery enrichment	[26]
*Alexandrium tamarense*	Saxitoxins; polyketides; fatty acids	Toxin production; ω-3 source	Diagnostic toxin kits; functional seafood supplements	[27]
*Haematococcus lacustris*	Astaxanthin	Antioxidant; skin and cellular protection	Supplements; seafood; pasta fortification	[28,29]
*Conticribra weissflogii*	Silica; LC-PUFAs	Biomaterial precursor; ω-3 source	Fortified foods; supplements	[29]
*Dunaliella salina*	β-Carotene	Provitamin A; immune and eye support	Dairy drinks; juices; margarine	[30]
*Phaeodactylum tricornutum*	EPA; fucoxanthin	Anti-inflammatory; antioxidant	Nutraceuticals; functional foods	[31]
*Dunaliella tertiolecta*	β-Carotene; glycolipids	Antioxidant; provitamin A	Natural colorant; nutraceuticals	[32]
*Arthrospira platensis* (*Spirulina*)	Phycocyanin	Antioxidant; immune-supporting	Smoothies; noodles; protein bars	[33]
*Phaeocystis pouchetii*	Polysaccharides; ω-3	Prebiotic; antioxidant	Prebiotic supplements; functional foods	[34]
*Nannochloropsis oceanica*	EPA; DHA; lutein	Antioxidant; eye health	Fortified foods; ω-3 supplements	[9]
*Chaetoceros calcitrans*	Carotenoids; silica	Antioxidant; ω-3 source	Nutritional supplements; food fortification	[35]
*Schizochytrium limacinum*	DHA; astaxanthin; betaine	Nutritional enhancement; antioxidant	Infant formula; beverages; bakery products	[36]
*Lyngbya majuscula*	Majusculamides; CYN	Antitumor; immunomodulatory	Functional ingredients; aquafeed	[37]
*Nannochloropsis oculata*	EPA	Anti-inflammatory; heart and brain health	Aquafeed; nutraceuticals; enriched eggs	[38]
*Nannochloropsis limnetica*	EPA; DHA; lutein; phycocyanin	Antioxidant; eye health	Functional foods; seafood colorant	[39]
*Tetraselmis chui*	DHA	Cognitive and visual development	Infant formula; dairy beverages	[40]
*Porphyridium purpureum*	ω-3 fatty acids; phycobiliproteins	Antioxidant; cardiovascular support	Supplements; natural food colorants	[41]
*Chaetoceros muelleri*	LC-PUFAs; fucoxanthin	Antioxidant; joint health	Supplements; fortified foods	[42]
*Tetraselmis suecica*	LC-PUFAs; polysaccharides	Prebiotic; lipid-lowering	Nutritional supplements; prebiotic additives	[40]
*Prymnesium parvum*	Polysaccharides; fatty acids	Immune-modulating	Nutraceuticals; diagnostic kits	[43]
*Skeletonema costatum*	LC-PUFAs; silica	ω-3 source; biomaterial precursor	Functional foods; supplements	[44]
*Thalassiosira pseudonana*	Silica; LC-PUFAs	Nutrient-rich biomaterial	Food fortification; supplements	[29]
*Chlorella vulgaris*	Chlorophyll	Detoxification; antioxidant	Dietary supplements; juices	[45]
*Haematococcus lacustris*	Astaxanthin	Anticancer; antioxidant	Aquafeed; cosmetics; nutraceuticals	[46]
*Tetradesmus obliquus*	Lutein; zeaxanthin	Eye health	Cereals; supplements; fortified foods	[47]
*Ankistrodesmus falcatus*	ω-3; polyphenols	Anti-inflammatory; antioxidant	Functional foods; supplements	[48]
*Botryococcus braunii*	Hydrocarbons; polysaccharides	Antioxidant; biofuel precursor	Algae proteins; biofuel feedstock	[49]
*Ecdysichlamys minuta*	Lutein; polysaccharides	Prebiotic; antioxidant	Nutraceuticals; functional foods	[50]
*Monoraphidium contortum*	ω-3; phycocyanin; polysaccharides	Immune-modulating; antioxidant	Nutritional supplements; algae proteins	[51]
*Chlorococcum infusionum*	Carotenoids; proteins	Protein-rich; antioxidant	Functional foods; protein products	[52]
*Kirchneriella lunaris*	PUFA; proteins; chlorophyll	Cardiovascular benefits	Supplements; functional foods	[53]
*Monoraphidium minutum*	β-Carotene; proteins	Provitamin A; protein source	Food coloring; algae proteins	[54]
*Desmodesmus subspicatus*	ω-3; polysaccharides	Heart health; prebiotic	Functional foods; supplements	[55]
*Aphanizomenon flos-aquae*	Phycocyanin; phenolics	Neuroprotective; anti-inflammatory	Dietary supplements; fortified foods	[56]
*Coelastrella* sp.	Proteins, carbohydrates, lipids, phenol, flavonoid	Antifungal, antioxidant properties, antimicrobial activity	Functional additives; stabilizers, functional food	[57]

**Table 3:** The compositional design and processing parameters of NADES systems.

Hydrogen-Bond Acceptor (HBA)	Hydrogen-Bond Donor (HBD)	Molar Ratios Used	Temp.	Ref.
Citric acid	monosaccharides (glucose, fructose, xylose, sorbose) Disaccharides (sucrose)	1:1 to 1:1:1 system; some mixtures diluted (e.g., 1:1:1:9) 1:1	~50 °C	[149,150]
Lactic acid	glucose or β-alanine	5:1; 1:1	~50 °C	[13,149]
Malic acid	glycerol, glucose, lactose, sucrose, xylitol, sorbitol, mannose	1:1; 2:5; 2:1; 1:1; 2:1; other binary systems	~50 °C	[13,149]
Betaine (Bet)	organic acids (citric, malic, oxalic) or sugars (glucose, sucrose, mannose)	1:1; 1:1:1 ternary mixtures; 2:1 to 5:2 systems	50–90 °C	[149]
D/L-Proline	sugars (sucrose, sorbitol, glucose) or organic acids (citric, malic, lactic, malonic)	Ratios ranging 1:1, 2:1, 3:1, 4:1; ternary mixes (e.g., 1:1:1)	~50 °C	[13,150]
Choline chloride (ChCl)	carboxylic acids (malonic, malic, oxalic, citric, acetic, butyric), sugars (glucose, xylose, xylitol, sucrose), polyols (glycerol, ethylene glycol), free fatty acids, phenyl acids	1:1, 1:2, 2:1, 2:5, 1:3 depending on donor	50–120 °C	[149,150]
	triethanolamine, zinc nitrate hexahydrate	1:2; 1:1		
N,N-Diethylethanolammonium chloride	malonic acid	1:1	~120 °C	[150,151]
DL-Menthol	acetic acid, lactic acid, pyruvic acid, lauric acid	1:1; 1:2; 2:1	~50 °C	[13,150]
Glycerol	amino acids (L-proline, L-alanine, glycine, L-histidine, L-threonine, L-lysine, L-arginine)	Typically, 3:1 or 4–5:1 (depending on amino acid)	≥100 °C	[149,150]

**Table 4:** Summary of key studies employing NADES-Assisted Green Extraction Strategies for the extraction of bioactive compounds from microalgae and other matrices.

NADES Composition	Molar Ratio	Biomass/Matrix	Extraction Technique	Optimized Conditions	Target Compound(s)	Yield/Recovery	Reference
Choline chloride:sugar	1:2	*Spirulina platensis*	UAE	60 °C; 20 min; solvent–biomass ratio 70 mL/mg	Pigments	~165 mg/g dry biomass;	[123]
Choline chloride-based DES	–	*Spirulina*	UAE	5% biomass; 40% amplitude; 22 min	Proteins	80.62% recovery; 442.88 mg/g extract	[183]
Choline chloride:urea	–	*Chlorella* sp.	UAE	1% biomass; 40% amplitude; 45 min	Proteins	32.79% recovery; 271.12 mg/g extract	[184]
Octanoic acid:decanoic acid	2.3:1	*Chromochloris zofingiensis*	UAE	50 °C; 49 min; LSR 66.2 mL/g	Canthaxanthin	70.4 µg/mL	[185]
Choline chloride:acetic acid	–	*Chlorella pyrenoidosa*	DES pretreatment + UAE	Pretreatment: 5 min; Extraction: MeOH:n-BuOH 1:4; 60 °C; 120 min; solvent–biomass 30:1 (*v*/*w*)	Lipids → FAMEs	19.25% lipid yield; biodiesel met EU/US standards	[186]
ChCl–oxalic acid/levulinic acid/urea/ethylene glycol/sorbitol	various	*Phaeodactylum tricornutum*	MAE (DES pretreatment + MW)	MW pretreatment; followed by DMC or scCO_2_ extraction	Total fatty acids (TFA)	+16% selectivity and +80% yield with DMC; 20-fold increase in scCO_2_ extraction	[15]
choline acetate ([Ch] [Ac])	-	*Nannochloropsis oceanica*	MAE	Temperature 40 °C; 30 min; 2% *w*/*v* IL; 0.5 g biomass	Proteins	26.35% yield (65.06% of total protein); Soxhlet = 0.63%	[187]
ChCl/Glycerol and ChCl/Urea	1:2	*Spirulina*	MAE	160 W; 1:40 biomass–solvent; 10 min	Proteins	30.48% yield; strong antioxidant and functional properties	[188]
ChCl/Glycerol and ChCl/Urea	1:2	*Chlorella*	MAE	160 W; 1:30 biomass–solvent; 10 min	Proteins	15.53% yield; selective anticancer activity	[188]
ChCl:glycerol/citric acid/urea/glucose	1:2	*Chlorella vulgaris* and *Scenedesmus incrassatulus*	MAE	500 mg of lyophilized biomass was put into 30 mL of NADES. Microwave oven (200 W), with a sonicator (24 KHz) at 60 °C for 20 min.	Phenolics, antioxidants, antibacterial compounds	Highest with ChCl-citric acid: TPC 4.98 mg/g; 75% DPPH; MIC 0.5 mg/mL (*S. aureus*)	[189]
ChCl–lactic acid	1:2	*Lippia citriodora*	MAE	63.68 °C, water content of 32.19%, 17.08 min	Bioactive compounds	Iridoids, 7.25 mg g^−1^, phenylpropanoids, 17.23 mg g^−1^, and flavonoids, 9.02 mg g^−1^	[190]
Beta-lactic acid	1:1	*Gnetum gnemon*	UAE	1:10 sample-to-solvent; 60% water; 10 min	Phenolic compound	0.3344 mg/g	[191]
Choline chloride–Lactic acid	1:2	Virgin olive pomace	MAE	Olive pomace (2 g) was mixed with 25 mL of NADES. Temperature 60 °C for 30 min duration	Phenolic compounds	9.4	[192]
ChCl–lactic acid	1:2	Virgin olive pomace	UAE	Olive pomace (2 g) was mixed with 25 mL of NADES. Temperature 60 °C, for 30 min, 60 kHz	Phenols	2.20	[192]
ChCl–lactic acid	1:3	*Cuminum cyminum* L. seed	MAE	cumin powder (20.0 g) at 110 °C, water content 40%, 5 min	Essential oil	1.57% (*w*/*w*)	[193]
ChCl–citric acid	2:1	Grape pomace	MAE	5 g of grape pomace were extracted using 500 mL NADES. 80 °C, 50 W, 10 min	Anthocyanins	1.77 mg gdw^−1^	[194]
Acetic acid–glycerol	2:1	Spent tea leaves	Simultaneous ultrasonic–enzymatic-assisted extraction (SUEAE), were performed using NADES	enzyme-added samples were simultaneously sonicated and hydrolyzed at 50 °C for 20 min, followed by enzymatic hydrolysis for 90 min. Each sample contained 0.5 g of spent leaves in 10 mL of NADES.	Total tannin contents (TTAC), total flavonoid content (TFC), and total terpenoid content (TTC)	179 (mg CE/g) TTAC, 17.3 (mg GAE/g) TFC, 157 (mg UE/g) TTC.	[195]

## Data Availability

All data supporting the findings of this study are included in the article.
